# Brief Strategy Training in Aging: Near Transfer Effects and Mediation of Gains by Improved Self-Regulation

**DOI:** 10.3390/brainsci12040465

**Published:** 2022-03-30

**Authors:** Carla M. Strickland-Hughes, Robin L. West

**Affiliations:** 1 Department of Psychology, University of the Pacific, Stockton, CA 95211, USA; 2 Department of Psychology, University of Florida, Gainesville, FL 32611, USA

**Keywords:** associative memory, memory self-efficacy, memory training, name recall, near transfer, strategy use

## Abstract

A common approach to cognitive intervention for adults is memory strategy training, but limited work of this type has examined intervention effects in relation to self-regulation (e.g., strategy usage, memory beliefs) and few have established near transfer (training-related performance gain on untrained tasks related to the target task). The present research, Everyday Memory Clinic—Revised (EMC-R), examined whether relatively brief face-name association training, offering elements focused on self-regulation, can improve name recall, enhance memory self-regulation, and lead to near transfer. Participants were 122 healthy, well-educated middle-aged and older adults (51–90 years old) randomly assigned to a strategy training program (SO), a comparable program with a theoretical self-regulatory boost (SB), or a waitlist control group. Compared to the waitlist group, both groups of trainees demonstrated higher pretest-posttest improvements in name recall (target task), memory self-efficacy, and effective strategy use, as well as the near transfer of gains to nontrained associative tasks, a rare finding in strategy training research. Furthermore, changes in memory self-efficacy and strategy use fully mediated the effect of training on name recall. This innovative approach for brief memory intervention offers a model for successful training that can be easily disseminated via community centers and lifelong learning programs.

## 1. Introduction

Everyday memory ability—such as learning and remembering names—is highly valued by people of all ages [1], yet is known to decline normatively with increasing age [2,3]. This decline is often accompanied by age-related vulnerabilities in related non-ability self-regulatory factors, such as self-evaluative beliefs and metamemory [4,5,6,7]. Fortunately, over six decades of research on episodic memory training confirm that older adults benefit from intervention: Trainees, compared to control groups, demonstrate improved performance on trained tasks immediately following training [8,9,10].

A traditional and common approach to memory training is teaching individuals mnemonics, or specific memory strategies, via didactic lectures, readings, videos, and practice exercises [9,11]. Meta-analyses and systematic reviews indicate that this type of training is effective for people who are over 60 years of age and that participation in cognitive interventions is better than doing no intervention activity [8,12,13]. However, the overall effectiveness of training varies across programs that differ along multiple dimensions, such as their design, format, duration, or content. For example, training might be more effective when it is longer, when it is conducted in groups, or when multiple strategies, rather than single strategies, are taught [8,12,14]. Furthermore, multifactorial programs—programs with non-strategy training components, such as attention or relaxation practice or information about how memory works—may be more effective than programs focused only on training strategies [12,15]. Although often untested, we propose these sorts of programs might be relatively more effective because they enhance self-regulatory factors, in addition to episodic memory [11,16]. Theoretically, flexible use of multiple strategies, including attention and selection of the best strategies after learning more about how memory works, may reflect self-regulatory choices while remembering [7,9,17,18].

Consistent with a recent self-regulatory focus, along with effective strategy selection and implementation [9,19,20], proposed mechanisms of training-related memory gains include improved self-evaluative beliefs [5,21,22]. Those most often studied include memory self-efficacy (confidence in the ability to perform defined memory tasks) and locus of control for memory (a sense that one can influence their own memory outcomes). Theoretical and empirical work is starting to suggest that self-regulation may be key to maximizing the impact of training on memory [16,23]. Indeed, self-evaluative beliefs are sometimes related to greater training-related improvements in cognition [22,24,25,26]. Beliefs are also related to concurrent memory performance [5] and predict memory performance up to six years later [27]. Results of a few studies suggest that self-regulatory factors might benefit from these programs and may also mediate or moderate training-related gains [25,26,28]. Thus, one purpose of this research was to highlight the value of self-regulation for training impact.

Furthermore, although memory training seems to produce immediate improvements in performance on trained episodic memory tests, controversy surrounds the true value of these programs in terms of the scope and duration of training-related gains [29,30]. Extensive debate surrounds the *what*, *when*, and *how* of transfer effects, wherein the training program results in meaningful improvements in skills or abilities that were not directly targeted in the training [31,32,33]. Controversial is the “generalist assumption”, suggesting that training gains are overstated because the benefits are limited only to the trained tasks, and only immediately following training [31]. As such, demonstrating transfer to untrained tasks has almost become a search for the “holy grail” of cognitive interventions.

Evidence for transfer from memory strategy training is so rare that some scholars have proposed it should be circumvented by directly training the tasks that older persons want to enhance [31], or by taking novel and alternative approaches to training, such as focusing on overall cognitive engagement [15,34,35] or practice of core competencies [32,33,36]. Indeed, the transfer can be observed in interventions that train core competencies, such as working memory or speed of processing, or provide extensive practice of such skills [24,32,37,38,39,40]. However, other scholars maintain that near transfer from memory strategy training is a reasonable goal, proposing that transfer might be facilitated by support, such as training monitoring skills [23,41,42], direct encouragement to apply learned strategies to new tasks [43], or offering training at home to promote transfer to “real world” memory [35]. Further, trainees with a growth mindset (similar to a greater sense of personal control) have exhibited transfer effects in working memory training [24]. Notably, those approaches emphasize metacognition, motivation, and other self-regulatory elements. Thus, enhanced self-regulation during training may theoretically facilitate the transfer of training benefits [11,23,34]. The present research was focused on both self-regulation and transfer issues.

### 1.1. The Present Study

An established, award-winning program—the Everyday Memory Clinic (EMC) [26]—was used as the foundation for this project. EMC is a multifactorial memory training program for adults over age 50. The design of EMC incorporated several elements to enhance memory self-efficacy across four known influences: enactive mastery, vicarious learning, verbal persuasion, and physiological and affective states [44,45]. Compared to waitlist control participants, EMC trainees demonstrated pretest-posttest gains in name recall, story recall, memory self-efficacy, memory control beliefs, and strategy use. Latent growth curve modeling [22] suggested that memory self-efficacy played a key role: Memory self-efficacy predicted performance levels, and gains in memory self-efficacy predicted gains in story recall. Thus, the EMC project was highly successful and highlighted the value of a focus on self-regulation in strategy training.

While successful, the EMC project was lengthy and intensive: Training covered multiple strategies for list, story, and name recall, lasted over five weeks, and included homework and readings outside of class [26]. Our partial replication aimed to extend EMC results by examining whether a brief training dosage, focused on one task, could produce similarly meaningful gains. Evidence suggests that relatively brief memory training programs can enhance performance on trained memory tasks [8,42,46,47]. While brief, our approach was still multifactorial (including attention and self-regulation elements) and included multiple strategies, both of which are important for successful training outcomes [8,15]. Thus, EMC-R was multifactorial, multi-strategy, and brief—an innovative format for training.

### 1.2. Research Aims and Hypotheses

#### 1.2.1. Aim 1. Impact of Training

Aim 1 was to determine whether multifactorial strategy training over one week could lead to significant pre–post improvements in recall and self-regulation, compared to a wait-list control group. In this project, EMC-Revised (EMC-R), multiple strategies were taught for name recall only, including attentional components, and training included one hour in class, with at-home exercises. Community-dwelling middle-aged and older adults were recruited, representing a wide age range. If qualified (see Methods), participants were randomly assigned to a waitlist control group or a one-week strategy training, with or without enhanced self-regulation elements. The primary outcome measures were name recall and self-regulatory factors, specifically memory self-efficacy, memory control beliefs, and strategy usage.

No differences in recall or self-regulatory factors were expected between the trained and control groups at pretest, due to random assignment. Practice effects were expected on name recall, such that all groups would have higher levels at posttest than pretest, as well as the percentage of strategies used. In contrast, memory self-efficacy can decline simply from testing and might show an overall decline from pretest to posttest. Our hypotheses (*HYP*) for Aim 1, as outlined here, consider the interaction effects for each outcome variable in turn. Trainees were expected to demonstrate greater pre-post improvements in name recall (*HYP1a*), memory self-efficacy (*HYP1b*), memory control beliefs (*HYP1c*), and percentage of strategies used (*HYP1d*), as compared to participants in the control group. Further, as assessed at posttest, a greater proportion of trainees than control participants were expected to report using the most effective name recall strategies (*HYP1e*).

#### 1.2.2. Aim 2. Impact of Added Self-Regulatory Elements

Secondarily, we investigated whether the enhancement of self-regulatory elements in training would lead to greater pre-post change than the basic multifactorial program (Aim 2). A secondary goal, Aim 2, was to determine whether the strategy plus enhanced self-regulation elements for one week (SB condition) demonstrated added value over the one-week strategy training-only condition without those additional elements (SO condition). We expected greater pre-post gains for SB compared to SO in name recall (*HYP2a*), memory self-efficacy (*HYP2b*), memory control beliefs (*HPY2c*), and percentage of strategies used (*HYPd*). These hypotheses were based on the idea that additional self-regulation elements would be motivational in nature and encourage more confidence and investment in training. We also examined whether SB, compared to SO, would lead to greater use of the effective name recall strategies (*HYP2e*).

#### 1.2.3. Aim 3. Near Transfer of Training Impact

Aim 3 was to determine whether EMC-R training could promote near transfer better pre-post gains for trainees (combined SO-SB) than the control group (CT) on untrained associative memory tasks.

We hoped to show that the impact of training could transfer to untrained associative memory tasks, an effect that is rarely shown in brief training. Enhanced self-regulation may serve as a key to maximizing the practical impact of training, by conveying to participants that personal performance change is possible, thus encouraging the transfer of training lessons to other tasks. We predicted higher scores for all groups on the posttest transfer tests, as compared to the pretest, due to practice effects. We hoped to find evidence of transfer on at least one of our untrained associative memory tasks (*HYP3*), due to the self-regulatory elements present in our training. However, given a dearth of evidence in past research, we did not expect to find substantial evidence for transfer. The transfer measures varied in modality and difficulty, with delayed and immediate recall/recognition tested for object-location (visual) and occupation-name (verbal) associations. Multiple outcomes were included to inform us whether *any* kind of transfer was possible, given the general failure to evidence near transfer in past memory strategy training.

#### 1.2.4. Aim 4. Mechanisms for Training Effects

Aim 4 was to test the mediation of training impact (that is, pre-post change in name recall performance) by those self-regulatory factors that showed greater pre-post change for trainees than the wait-list control group.

The project was designed to examine the power of self-regulation variables to mediate training-related performance change, an innovative approach in training research. Considering that strategy training has been implemented for decades, it is especially surprising that direct examination of strategy usage as a mediator for performance change is rare. However, strategy training across decades has resulted in significant changes in memory performance. Thus, we expected that memory change would be governed in part by strategy change. Theoretically, both self-efficacy and memory control beliefs are related to motivation and effort and could impact the level of performance change as a function of training. Thus, any of our self-regulatory factors that show greater pre-post change for trainees than controls could mediate pre-post change in name recall performance (*HYP4*).

## 2. Materials and Methods

### 2.1. Participants

Community-dwelling middle-aged and older adults were recruited from the Gainesville, Florida area using newspaper advertisements, participant pools, lifelong learning programs, and word-of-mouth. Participants paid a fully refundable deposit of $25 to receive the training materials, valued at $45. For compensation, participants who completed the research interviews were refunded the full deposit and kept all training materials at no additional cost.

Inclusion criteria for the study were vision, hearing, and English skills adequate to complete the interviews and training, freedom from cognitive impairment, no reported major stroke or head injury in the previous year, more than eight years of education, and an age of at least 50 years. Of the 130 eligible participants, 8 were later excluded for inability to follow instructions during assessments. The final sample included 122 participants, and 117 completed the entire research program (one dropped for health reasons; two in SO and two in SB quit for unspecified reasons).

Participants were 51 to 93 years old (*M* = 73.24 years, *SD* = 8.31 years), with half under 75 years. Most were female (79%), Caucasian (92%), healthy, and well-educated (years of education ranged from 11 to 27, with *M* = 17.33 years, *SD* = 2.84 years). Self-reported health was assessed with the SF-12 [48]. Norm-based SF-12 standardized scores for physical and mental health have means of 50 and standard deviations of 10 in the general U.S. population. Scores for physical health in this sample ranged from 18 to 64 (*M* = 49.04, *SD* = 9.35), and scores for mental health ranged from 26 to 65 (*M* = 54.68, *SD* = 6.58). Additional details on participants can be found in Appendix A.

Recruitment of this sample size was informed by a priori power analyses conducted in G*Power (version 3.0.10; [49], and the final sample size was consistent with those used in previous research [26,50]. The main design of the research was a 2 (time: pretest, posttest) × 2 (training condition: CT vs. combined SO-SB; *OR* SO vs. SB) mixed-model design with time as a within-subject variable and training condition as a between-subject variable. Relevant effect sizes from the EMC project were large [26]. The power derived from this sample size was sufficient to detect medium-large effect sizes in the analyses (e.g., repeated measures ANOVAs with two variables and two groups, with between-repetition correlations for outcomes estimated as *r* = 0.40–0.80, based on memory self-efficacy and name recall data from previous studies [51].

### 2.2. Procedure

Completion of the research program for each participant (i.e., pretest, training, posttest) lasted three weeks (see Table 1). All participants completed an initial telephone interview of 30–45 min, which started with verbal informed consent, and week 1 pretest and week 3 posttest interviews of approximately 2 h. All study measures are listed in order of administration in Table 2.

Pretest and posttest assessments were administered in small group sessions (*n* = 1–13). Across all testing and training sessions, the mean group size was 5.56 participants (*SD* = 3.63 participants). Procedures for the pretest and posttest assessments were nearly identical. However, alternative versions of the memory tasks using different stimuli (see Supplemental Materials) were administered, counterbalanced by time and condition. The pretest assessment began with written informed consent, followed by administration of paper-and-pencil tasks, with research assistants nearby to ensure that all instructions were followed. Testing sessions included participants from all conditions, to ensure that no individual tested better due to the presence of others from their training class.

### 2.3. Measures and Materials

#### 2.3.1. Baseline, Control, and Exploratory Measures

Participant eligibility, baseline ability factors, and other individual factors were assessed in an initial telephone interview. Most of these factors were later used to evaluate whether random assignment by block resulted in comparable groups (see Results). Details on these measures are explained in Supplementary Materials A, and these measures did not differ significantly across the condition assignment (see Results). Supplementary Materials A also has descriptions of a few exploratory measures that were administered after the primary outcome measures.

#### 2.3.2. Primary Outcome Measures

Primary outcome measures were assessed at the pretest and posttest, including name recall performance on the face–name association memory task (the focus of training), and self-regulatory factors that were identified as related to episodic memory: memory self-efficacy, memory control beliefs, and strategy use.

**Name recall.** The name recall assessment adopted EMC procedures and materials [26]. Participants completed two levels of the name recall task. Level 1 of the task included 12 name-face pairs and level 2 added 12 more, for a total of 24 name-face pairs. At each level, testing immediately followed intentional encoding, with a 1-min study and 2-min recall for level 1, and 5-min study and 5-min recall for level 2. The to-be-remembered stimuli were names printed in all capital letters underneath color portraits of ethnically diverse men and women of all ages (see Supplementary Materials B). During testing, grayscale versions of the same portraits were presented in a different order, without names, and participants were asked to write down each person’s name under the face. The level 1—level 2-tiered structure allowed for the maximal influence of self-regulatory factors by allowing individuals to invest additional effort over time on the second trial, if willing to do so, and the primary dependent measure was percent correct on level 2 (see additional details in West et al. [26]).

Two sets of name–face pairs were used, having similar balances of race, gender, and age, and matched difficulty based on past research [26]. The set assignment was counterbalanced by the testing occasion (pretest, posttest) and training conditions. Name recall performance did not vary with use of a particular set of name–face pairs at pretest for level 1, *t*(119) = −1.34, *p* = 0.182, or for level 2, *t*(119) = −0.89, *p* = 0.375, or at posttest for level 1, *t*(115) = −0.56, *p* = 0.574, or at level 2, *t*(115) = 0.032, *p* = 0.975.

**Memory Self-Efficacy.** Memory self-efficacy represents an individual’s level of confidence in the ability to perform defined memory tasks. Following well-established procedures for the Memory Self-Efficacy Questionnaire-4 (MSEQ-4) [56], respondents indicated their confidence (from 0 = *I cannot do it* to 100 = *100% sure I can do it*) in remembering names, object locations, shopping lists, and stories (with five levels of difficulty for each task). Confidence was averaged across all 20 items to calculate one memory self-efficacy score (range: 0–100). As in past research, the MSEQ-4 had very good internal consistency (pretest Cronbach’s α = 0.93, posttest Cronbach’s α = 0.93).

**Locus of control for memory.** Memory control beliefs were assessed using the well-known Locus subscale of the Metamemory in Adulthood Questionnaire (MIA) [57]. This subscale assesses individuals’ perceived sense of control over their memory skills, e.g., *If I were to work on my memory, I could improve it*. Responses across nine items (range: 1–5) were averaged and a higher score indicated greater perceived control over memory. The Locus subscale had adequate internal consistency reliability (pretest Cronbach’s α = 0.73, posttest Cronbach’s α = 0.73), comparable to past research [56].

**Name recall strategy use.** Greater strategy use indicates greater self-regulation because it shows that participants flexibly employed multiple strategies. Using procedures from the EMC project [26], a 16-item strategy checklist was administered following level 2 name recall testing. Example strategies included *I tried to pick out prominent features* and *I repeated names over and over to myself*. Participants checked each memory strategy that they used and wrote in additional strategies, if they wanted (any unique strategies were counted). At the bottom of the checklist, participants identified one or two methods used “most often for remembering”. The strategy checklist is reproduced in Supplementary Materials C.

One dependent variable was percentage strategy usage, based on the number of strategies selected from the checklist. As a second indicator of strategy usage, we analyzed the proportion of trainees in each condition who “most often” employed the most effective strategies. The most effective strategies were identified by comparing name recall performance between participants who did and did not report using each of the named strategies on the checklist.

#### 2.3.3. Transfer Outcome Measures

Given the limited near transfer reported for past memory strategy training programs, four different untrained associative memory tasks were administered at pretest and posttest to permit a broad and nuanced examination of potential transfer—object–location visual association and occupation–name verbal association. These measures were adapted from similar paradigms reported in the literature, but test timing and difficulty were fine-tuned with pilot testing. Two sets of items for each task were developed and counterbalanced across testing occasions and training conditions. Post hoc paired *t*-tests confirmed that the different sets were of comparable difficulty; performance did not vary between the two sets at pretest or posttest for any of the transfer measures (all *p*s > 0.05). As with name recall, strategy checklists for each association task were completed immediately following testing (see Supplementary Materials C)

**Object-location association (visual).** The object-location task was adapted from West, Welch, and Knabb [60]. Location recall was assessed using a matrix array wherein participants placed 24 pictures of objects (e.g., hat, scissors) in a 3 × 4 array representing 12 rooms in a house. A complete list of the objects, example pictures of objects, and an example room array are included in Supplementary Materials D. Participants were given five minutes to place the objects and to study their placements (with no more than two objects permitted in each room). At delayed recall (about 40 min later), participants had five minutes to reconstruct their arrays. Arrays were photographed by research assistants after immediate placement and after the delayed recall. Comparing photographs, object-location scores were calculated as the percentage of objects placed in the same room at encoding and retrieval.

**Occupation-name association (verbal).** The occupation-name task assessed immediate recall, delayed recall, and delayed recognition memory for names paired with occupations. Two different sets of 30 occupation-name pairs were used, with name stimuli from past research [58,59]. Within each set, half of the occupations and half of the names were concrete and could be visualized (e.g., FARMER, STONE). The two sets were matched by the length of the name (letters and syllables) and frequency of names (per data published by the United States Census Bureau from the 2000 Census), as well as a general familiarity with the occupations and ease of visualization, per ratings from an independent sample (*N* = 14).

Adapting procedures from past research [43], participants were given six minutes to study 30 pairs of names and occupations written in capital letters on laminated 4″ × 1.5″ index cards. At immediate and delayed recall, participants were given four minutes to record paired names for a list of 30 target occupations (two different orders were used for immediate and delayed recall), and the percentage of correctly matched names was recorded. Immediate recall was tested right after encoding, delayed recall after 40 min, and delayed recognition about 75 min after encoding. During recognition, participants had three minutes to review 30 familiar occupations and names, but only half were paired correctly. The percentage of correct hits (“Yes” circled for correct pairs) and correct rejections (“No” circled for incorrect pairs) was calculated. A complete list of the occupation–name stimuli is included in Supplementary Materials E.

### 2.4. Training Procedures

Participants were scheduled to attend testing sessions based on availability. Research participants were then randomly assigned by session “block” to one of three training conditions: a waitlist control group (CT; *n* = 38), strategy training (SO; *n* = 46), or strategy training plus self-regulatory elements (SB; *n* = 38). Assignment ensured adequate numbers for each class (*n* = 12–20, based on the Everyday Memory Clinic).

#### 2.4.1. Overall Training Approach

All trainees (SB and SO) completed a two-hour group class the second week of the research program, including association and visualization strategies for names paired with faces, such as the image-name match method, as well as attentional techniques. The content and structure of the class were consistent with those reported by West and colleagues [26]. The image-name match method involves several steps, with the goal of visualizing an object that represents the person’s name (e.g., can for Ken; a dollar bill for Bill) next to an exaggerated version of a prominent or distinctive facial feature. The instructor explained each step in all recommended strategies, and modeled strategy use. Then trainees practiced each technique in class, e.g., each trainee came up with an association for their own name and shared this with the group. Training duration, strategy instruction, and practice exercises were the same for SB and SO. CT received no training. Classes were video-recorded to establish quality control and consistency across multiple sessions.

At the end of the training class, participants received a workbook with a written transcript of the training content, readings on name recall strategies, and practice exercises for face recognition, name recognition and recall, and name-face association. All trainees were asked to complete two to three hours of self-study with these materials in the same week as the training class but before the posttest. Trainees were asked to document their work (see Supplementary Materials F). Trainees received lab contact information, in case of questions about the homework, but no participants contacted the research team.

#### 2.4.2. Self-Regulatory Elements in Training

Although the strategies taught did not differ by training group, the SB training contained additional elements designed to foster self-regulation, based on Bandura’s self-efficacy theory. Four influential sources of self-efficacy were targeted: enactive mastery, vicarious experience, verbal persuasion, and physiologic and affective states [44,45]. As shown in Table 3, the SO and SB classes both supported self-regulation, but more support was provided to the SB group. Elements that might facilitate self-regulation in the SO condition included modeling of the trained strategies by the instructor or class members, social support or comments by class members about the usefulness of strategies, recognition by class members that most people have memory problems, extensive practice, and personal feelings of mastery over strategy content. Group training situations cannot prevent peer influence.

In the SB groups, however, the instructor and the workbook focused on more self-regulatory elements. The SB training emphasized “personal learning,” rather than getting 100% correct, and SB trainees were asked to set their own goals for practice and learning. Additionally, the practice exercises in the SB workbook were in order of increasing challenge with simpler exercises first, rather than random. SB trainees also received explicit positive feedback from the instructor and heard the instructor reframe negative comments to ensure all trainees that they could accomplish their goals. All these elements had been included in EMC, with a longer, more intensive training course, but our purpose here was to see if a single week course with fewer elements (SO) would be effective, or if greater training impact would occur only with more self-regulatory elements (SB).

Table 3 shows the self-regulatory elements in SB and SO and how they are theoretically related to self-efficacy. Written workbooks for SB and SO also varied (see specific written examples in Appendix B). Five expert raters compared the written materials for SO and SB, looking for the listed elements. The intraclass correlation coefficient for their ratings was 0.95. The SB written training materials (*M* = 140.40, *SD* = 14.15) had significantly more incidences of self-efficacy support elements than the SO written training materials (*M* = 95.20, *SD* = 9.65), *t*(4) = 12.77, *p* < 0.001.

## 3. Results

The research employed a mixed-model design for examination of research aims via 2 within (time) × 2 between (condition) repeated measures analyses of variance (ANOVAs) and χ^2^ tests of independence. Reported effect sizes are partial eta-squared (η^2^) values from SPSS versions 24 and 27 [63]. With the unbalanced cell sizes, Pillai’s Trace *F*-approximations are reported for all repeated measures ANOVAs with a significant Box’s *M* test of homogeneity of covariance (*p* < 0.001). Furthermore, none of the Levene’s test statistics for any outcome variable were significant, indicating the assumption of homogeneity of variances was met for each analysis. All pairwise post hoc comparisons were conducted with Bonferroni-corrected analyses, and *p*-values are reported where significant. An alpha level of 0.05 was used as the significance criteria.

We reported the results of the mixed model ANOVAs to evaluate both level and change in our outcome measures. However, as an alternative analytic approach, we conducted a series of general linear models wherein posttest performance was compared for the training and control groups with pretest performance included as a covariate. Our pattern of results was unchanged (details available upon request).

### 3.1. Preliminary Results: Baseline Data by Condition

At baseline, univariate analyses showed that CT, SO, and SB groups did not differ in years of education, subjective ratings of English (speaking, writing, and comprehension), vision, or hearing, performance on tests of episodic memory (list recall) and working memory (digits backwards), self-reported physical and mental health, global perceived mastery, or general memory ratings. Pearson’s χ^2^ tests confirmed that random assignment was independent of gender. All preliminary measures are described in Supplementary Materials A, and means and standard deviations by condition appear in Appendix C.

However, before testing, the groups varied by age, *F*(2, 121) = 3.50, *p* = 0.033. Follow-up pairwise comparisons confirmed that the SB participants were older than the SO participants (*M*_diff_ = 4.57, *SE* = 1.78, *p* = 0.035) but not the CT participants (*M*_diff_ = 3.56, *SE* = 1.87, *p* = 0.176), and the SO and CT participants did not differ in age (*M*_diff_ = 1.01, *SE* = 1.78, *p* = 1.00). To address this pre-existing difference, all analyses were conducted with and without age included as a covariate. Because the pattern of results was the same, the analyses are reported without age as a covariate.

### 3.2. Aim 1 Results: Impact of Abbreviated Training

The first aim of the present research was to test whether our brief training was effective. Separate 2 time (within: pretest, posttest) × 2 condition (between: CT, trained SO-SB) repeated measures ANOVAs were conducted for name recall level 2, memory self-efficacy, memory control beliefs, and percentage of strategies used. A Pearson’s χ^2^ test was conducted for the categorical outcome variable (whether effective strategies were used). Observed means and standard deviations are reported in Table 4.

#### 3.2.1. Training Effects for Name Recall

**Figure 1:**
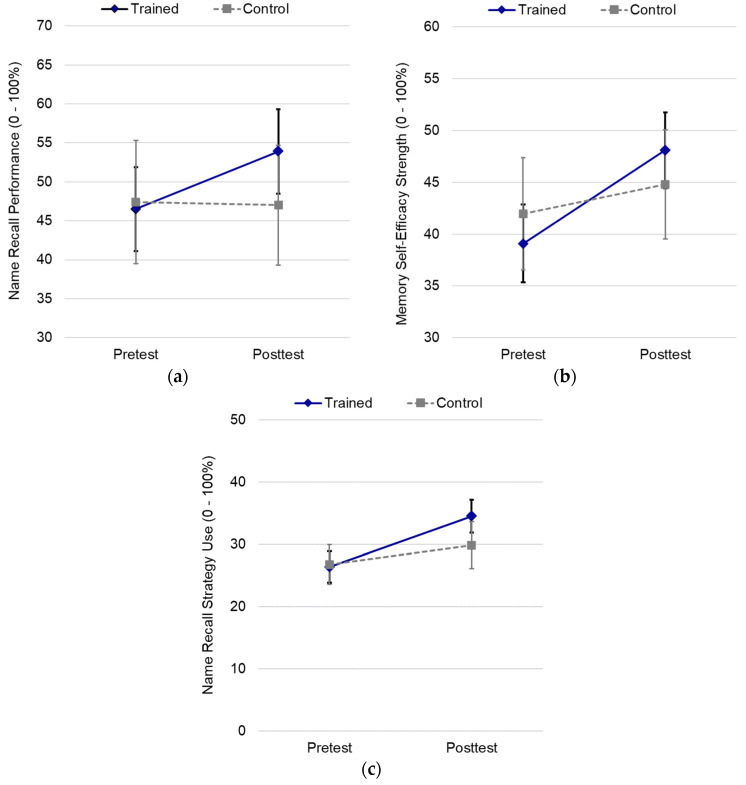
Effect of brief training on primary outcome measures: (**a**) name recall performance; (**b**) memory self-efficacy; and (**c**) name recall strategy use. Error bars depict 95% confidence intervals.

As expected, the main effect of time was trend-wise significant, *F*(1, 115) = 3.61, *p* = 0.060, η^2^ = 0.03, with a higher performance at posttest than pretest. Qualifying this main effect, the interaction between time and condition was significant, *F*(1, 115) = 4.32, *p* = 0.040, η^2^ = 0.04. Post hoc pairwise comparisons indicated that performance between trainees and control participants did not differ at pretest or at posttest. However, confirming *HYP1a*, trainees performed better at posttest than they did at pretest (*M*_diff_ = 7.33, *SE* = 2.10, *p* < 0.001), whereas control participants did not (*M*_diff_ = −0.33, *SE* = 3.03, *p* = 0.914), as shown in Figure 1a.

#### 3.2.2. Training Effects for Memory Self-Efficacy

Unexpectedly, memory self-efficacy was higher overall at posttest than at pretest, *F*(1, 115) = 27.62, *p* < 0.001, η^2^ = 0.19. This main effect for time was qualified by a significant interaction between time and condition, *F*(1, 115) = 7.51, *p* = 0.007, η^2^ = 0.06, consistent with *HYP1b*. Post hoc pairwise comparisons indicated that memory self-efficacy between conditions did not differ at pretest or at posttest, but trainees reported greater memory self-efficacy at posttest than they did at pretest (*M*_diff_ = 8.99, *SE* = 1.28, *p* < 0.001), whereas control participants showed no change (*M*_diff_ = 2.83, *SE* = 1.85, *p* = 0.129), as shown in Figure 1b.

#### 3.2.3. Training Effects for Memory Control Beliefs

The main effect of time was significant for memory control beliefs, *F*(1, 115) = 7.10, *p* = 0.009, η^2^ = 0.06, which were higher at posttest than at pretest across both conditions. The interaction between the time and condition, however, was not significant, *F*(1, 115) = 2.29, *p* = 0.133, η^2^ = 0.02, which did not support *HYP1c*.

#### 3.2.4. Training Effects for Strategy Use

**Percentage of strategies used.** Consistent with expectations, the main effect of time was significant for the percentage of strategies used, *F*(1, 115) = 26.72, *p* < 0.001, η^2^ = 0.19, with more strategies reported at the posttest than pretest. Further, the interaction between the time and condition was significant, *F*(1, 115) = 5.70, *p* = 0.019, η^2^ = 0.05, in support of *HYP1d.* Post hoc pairwise comparisons indicated that the strategy use did not differ between trainees and control participants at pretest (*M*_diff_ = −0.64, *SE* = 1.98, *p* = 0.746), but trainees used a greater number of strategies than control participants at posttest (*M*_diff_ = 4.67, *SE* = 2.33, *p* = 0.047). Control participants reported no pre–post change in strategy use (*M*_diff_ = 3.10, *SE* = 1.83, *p* = 0.093), but trainees reported pre–post gains in strategy use (*M*_diff_ = 8.41, *SE* = 1.27, *p* < 0.001). The significant interaction is depicted in Figure 1c.

**Effective strategy use.** Effective name recall strategies were operationalized as strategies that were associated with higher percent correct at level 2 in independent samples *t*-tests. The two-tailed significance levels of α = 0.05 were used, although a priori expectations were directional (the use of a given strategy was expected to be related to better performance than not using the strategy). Chi-square tests were then used to examine whether the most effective strategies were reported more often by the CT or trainee groups.

***Identification of effective strategies.*** The most effective strategy at both pretest and posttest was the “familiar name” strategy (i.e., *I associated the name with the name of someone else I know*). Participants who reported using the familiar name strategy, compared to those who did not, performed significantly better at pretest (48% used; *M*_diff_ = 14.91, *SE* = 4.27), *t*(119) = 3.49, *p* < 0.001, and at posttest (54% used; *M*_diff_ = 16.45, *SE* = 4.21), *t*(116) = 3.90, *p* < 0.001.

In addition, participants who reported using the “associate name” strategy (i.e., *I tried to think of a meaningful association for the name*) performed significantly better than those who did not at pretest (57% used; *M*_diff_ = 10.76, *SE* = 4.41), *t*(119) = 2.44, *p* = 0.016, and use of the associate name strategy was marginally related to better performance at posttest (65% used; *M*_diff_ = 8.35, *SE* = 4.62), *t*(116) = 1.81, *p* = 0.073.

Two other strategies were effective at pretest or posttest, but not both. Participants who reported using the “familiar face” strategy (i.e., *I associated the face with the face of someone else I know*) performed comparably to those who did not at pretest (37% used; *M*_diff_ = 5.15, *SE* = 4.61), *t*(119) = 1.12, *p* = 0.266, yet had superior performance at posttest (50% used; *M*_diff_ = 13.77, *SE* = 4.28), *t*(116) = 3.22, *p* = 0.002. Participants who reported using the “self-test” strategy (i.e., *I covered the faces, looked away and tested myself on the names*) performed better than those who did not at pretest (14% used; *M*_diff_ = 17.74, *SE* = 6.24), *t*(119) = 2.84, *p* = 0.005, but this benefit was not evidenced at posttest (29% used; *M*_diff_ = 5.59, *SE* = 4.90), *t*(116) = 1.16, *p* = 0.248.

The two strategies that were generally effective (familiar name and associate name), as well as the one effective only at posttest (familiar face), involved association. Forming associations was a focus of the training class so these three strategies were considered as an effective associative strategy set. The self-test strategy showed no benefit on the posttest, so it was not examined further.

***Examination of effective strategy use by training condition.*** Chi-square tests of independence were conducted to evaluate whether effective strategy use varied between trainees and control participants. At pretest, trainees and control participants did not differ in their use of the associative strategy set, χ^2^(1) = 1.36, *p* = 0.244. However, consistent with *HYP1e*, there was a significant relationship between training and use of the associative strategy set at posttest, χ^2^(1) = 5.08, *p* = 0.024. Based on the odds ratio, the odds of participants preferring one of the effective association strategies at posttest were 2.48 times higher if they received training (combined SB-SO) than if they did not receive training (CT).

### 3.3. Aim 2 Results: Impact of Added Self-Regulation Elements

Secondarily, Aim 2 addressed the possibility that the added self-regulatory elements in condition SB would lead to greater training impact than condition SO. Separate 2 time (within pretest, posttest) × 2 condition (between SO, SB) repeated measures ANOVAs were conducted for name recall, self-efficacy, control, and percentage of strategies used. Means and standard deviations are reported in Table 5. A Pearson’s χ^2^ test was conducted to examine the usage of the effective associative strategy set. The main effects for time were consistent with the pretest-posttest results for the training group in Aim 1 results. Our focus here was on time × condition interactions.

For name recall, pre–post improvement in name recall performance was not greater for SB trainees than for SO trainees (*HYP2a*), as indicated by a non-significant interaction effect, *F*(1, 77) = 0.03, *p* = 0.860, η^2^ < 0.001. In fact, none of our hypotheses about greater training change for SB than SO were confirmed, with no significant time by condition interactions for memory self-efficacy (*HYP2b*), *F*(1, 77) = 1.04, *p* = 0.310, η^2^ = 0.01, memory control beliefs (*HYP2c*), *F*(1, 77) = 1.18, *p* = 0.280, η^2^ = 0.02, or strategy use *(HYP2d*), *F*(1, 77) = 1.33, *p* = 0.831, η^2^ < 0.01. Chi-square tests of independence also showed no differences between the groups at pretest, χ^2^(1) = 0.27, *p* = 0.606, or at posttest, χ^2^(1) = 0.45, *p* = 0.503, in reported preferred use of the effective strategy set (*HYP2e*).

### 3.4. Aim 3 Results: Near Transfer Effects

The third aim of this research was to determine whether our brief training program would promote near transfer, as evidenced by pre-post performance gains on untrained associative memory tasks.

#### 3.4.1. Preliminary Analyses for Transfer Outcomes

The four transfer outcomes were expected to be related to each other and to the targeted name recall task, but to represent distinct memory performance variables. Bivariate correlations among the transfer outcomes ranged from *r* = 0.34 to *r* = 0.97, with the strongest relationships between the immediate and delayed occupation-name recall tests. Relationships between level 2 name recall performance and the transfer outcomes ranged from *r* = 0.46 to *r* = 0.62. The correlations overall suggest that the transfer tasks were not highly related to the name recall task, but that training-related improvements on the occupation–name recall tasks might represent more proximal transfer than improvements on the occupation–name delayed recognition task or the object-location recall task. Pearson’s *r* correlation coefficients are reported in Appendix D. All correlation coefficients were significant (*p* < 0.001). Analyses compared CT vs. SB-SO trainees. Comparisons between SB and SO trainees were not made for the transfer outcomes as there were no training differences between these groups. Observed means and standard deviations for all four transfer outcomes by training condition at pretest and posttest are summarized in Table 6.

#### 3.4.2. Near Transfer Effects

**Figure 2:**
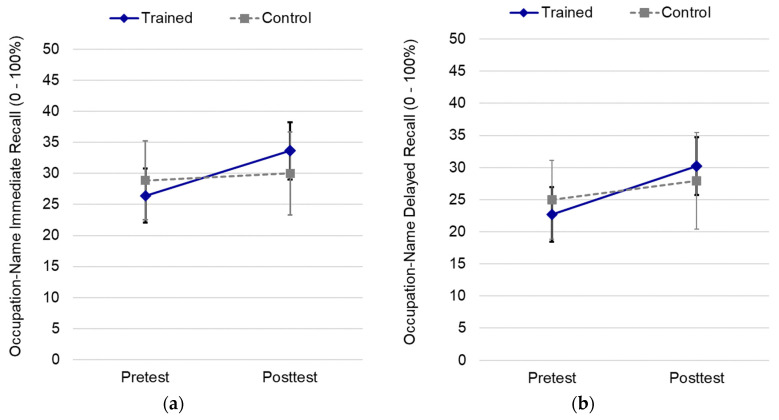
Near transfer effects of brief training for: (**a**) occupation–name immediate recall; (**b**) occupation-name delayed recall. Error bars depict 95% confidence intervals.

**Occupation-Name Immediate Recall.** Near transfer to occupation-name immediate recall showed a significant main effect of time, *F*(1, 116) = 8.88, *p* = 0.004, η^2^ = 0.07, and the interaction between time and condition was significant, *F*(1, 116) = 4.69, *p* = 0.032, η^2^ = 0.04. Performance was better at posttest than pretest for SB-SO trainees (*M*_diff_ = 7.21, *SE* = 1.59, *p* < 0.001, 95% CI [4.06, 10.36]), whereas the control group demonstrated no pre–post change in performance (*M*_diff_ = 1.14, *SE* = 2.31, *p* = 0.622, 95% CI [−3.43, 5.71]), see Figure 2a.

**Occupation-Name Delayed Recall.** Near transfer to occupation–name delayed recall was also tested. Like other transfer measures, the mean performance was greater at posttest than at pretest, *F*(1, 116) = 12.21, *p* < 0.001, η^2^ = 0.10. The interaction between time and condition was also significant, *F*(1, 116) = 4.26, *p* = 0.041, η^2^ = 0.04. Performance was better at posttest than pretest for SB-SO trainees (*M*_diff_ = 7.508, *SE* = 1.53, *p* < 0.001, 95% CI [4.47, 10.53]), whereas the control group demonstrated no pre–post change in performance (*M*_diff_ = 1.93, *SE* = 2.22, *p* = 0.387, 95% CI [−2.47, −6.33]), as shown in Figure 2b.

**Occupation-Name Delayed Recognition.** The main effect of time was significant for occupation name delayed recognition, *F*(1, 116) = 12.11, *p* < 0.001, η^2^ = 0.10. Overall, mean performance was greater at posttest than at pretest, but the interaction between time and condition was not significant, *F*(1, 116) = 1.79, *p* = 0.184, η^2^ = 0.02.

**Object-Location Recall.** Neither the main effect of time, *F*(1, 110) = 0.04, *p* = 0.844, η^2^ < 0.001, nor the interaction between test and condition, *F*(2, 110) = 0.05, *p* = 0.825, η^2^ < 0.001, were significant for object location recall. Performance on this task did not improve as a function of training or repeated testing.

For all of the occupation name measures, but not for the object locations tests, the expected practice effects were observed. The pretest-posttest gains were evidenced for the training groups, but not for the waitlist control group, for the immediate and delayed assessment of occupation–name associative memory, consistent with *HYP3*.

### 3.5. Aim 4 Results: Mechanisms for Training Effects

**Figure 3:**
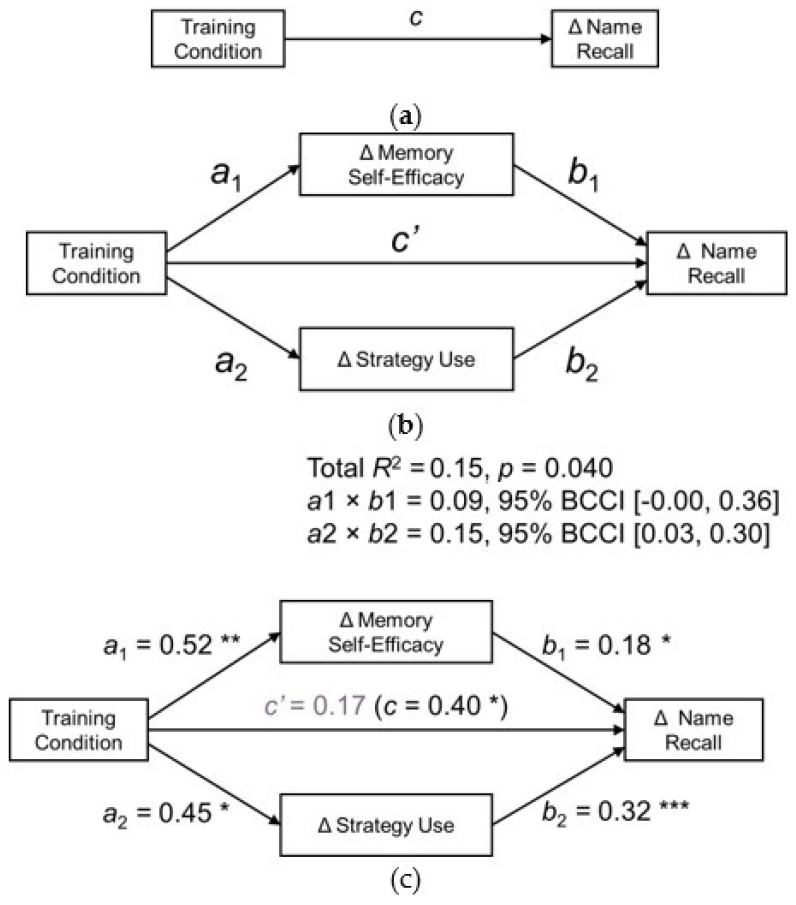
Multiple mediation model testing the mediation effect of self-regulatory factors on the impact of memory training on name recall of brief training on primary outcome measures: (**a**) Direct effect of training condition (waitlist control versus training) on change in name recall performance; (**b**) hypothesized path model, (**c**) multiple mediation model and coefficients. All reported coefficients are unstandardized. Δ: Standardized pretest–posttest change scores; BCCI: bias-corrected confidence interval. * *p* ≤ 0.05, ** *p* ≤ 0.01, *** *p* ≤ 0.001.

The fourth aim of this research was to test whether training-related gains in name recall performance would be mediated by self-regulatory factors. As summarized in the Aim 1 results, in addition to having greater training-related gains in name recall memory performance, trainees—but not waitlist control participants—demonstrated pretest-posttest gains in both memory self-efficacy strength and strategy use. Thus, we conducted a multiple parallel mediation model using INDIRECT SPSS Macro for Multiple Mediation (version 3.0) [64] to assess whether the effect of training on name recall was mediated by change in the self-regulatory variables of memory self-efficacy and strategy use. The training condition served as the independent variable in the mediation model, with 0 = CT and 1 = SO-SB. The dependent variable was the standardized pretest-posttest change in name recall memory performance. The parallel mediators were the standardized pretest-posttest change in memory self-efficacy and standardized pretest-posttest change in the percentage of strategies used on the name recall task. The use of standardized change scores for mediators allowed us to evaluate the role of self-regulatory gains relative to the average pretest–posttest change. Testing the mediators in parallel also allowed for a comparison of their relative influence on the change in name recall. Five thousand bootstrapped samples were generated. Unstandardized coefficients and 95% bias-corrected confidence intervals (BCCI) were reported. Figure 3 shows the hypothesized path model and results.

The omnibus test of total effect was significant, *F*(1, 113) = 6.67, *p* = 0.040, *R*^2^ = 0.15. Overall, the model explained 15% of the variance in the standardized pretest–posttest change in name recall performance. The total effect (*c* path; direct and indirect effects) of training on name recall was significant (*b*-weight = 0.40, *se* = 0.19, *p* = 0.040, 95% BCCI [0.02, 0.79]). However, after accounting for the impact of the indirect paths through the two mediators, (both significant), the direct effect of training on name recall (*c*′ path) was no longer significant (*b*-weight = 0.17, *se* = 0.19, *p* = 0.007, 95% BCC [−0.22, 0.55]). This finding suggests that the training effect on name recall memory was fully mediated by pretest-posttest change in self-regulatory factors.

The training was related to the greater standardized change in memory self-efficacy, which in turn was related to the greater standardized change in name recall performance. For the standardized change in memory self-efficacy, the effect of training (*a*1 path) was significant (*b*-weight = 0.52, *se* = 0.19, *p* = 0.007, 95% BCCI [0.14, 0.90]), suggesting that the training group reported about half a standard deviation greater pretest–posttest improvement in memory self-efficacy than did the control group. Then, the relationship between memory self-efficacy and name recall (*b*1 path) trended towards significance (*b*-weight = 0.18, *se* = 0.09, *p* = 0.054, 95% BCCI [−0.00, 0.36]). Thus, the *a*1 × *b*1 indirect effect was significant with a point estimate of 0.09, 95% bootstrapped BCCI [0.00, 0.25].

A similar pattern was observed for the indirect path through change in strategy use: The effect of training (*a*2 path) was significant (*b*-weight = 0.45, *se* = 0.19, *p* = 0.019, 95% BCCI [0.08, 0.82]). The training group reported almost half a standard deviation greater pretest–posttest improvement in strategy use than did the control group. Change in strategy use was positively related to change in name recall performance (*b*-weight = 0.32, *se* = 0.09, *p* = 0.001, 95% BCCI [0.14, 0.51]). The *a*2 × *b*2 indirect effect was significant with a point estimate of 0.15, 95% bootstrapped BCCI [0.03, 0.30].

Contrast testing suggested that the weight of the two indirect effects might be comparable, 95% bias-corrected confidence interval [−0.23, 0.13]. This evidence for a full mediation of the effect of training on name recall performance through the change in memory self-efficacy and change in strategy use is consistent with *HYP4*.

## 4. Discussion

Evidence from the present research advances our understanding of the practical impact of memory strategy training in midlife and beyond with its focus on memory self-regulation and near transfer. Overall, this research demonstrated that brief name recall strategy training is effective for middle-aged and older adults. The training program constituted two hours of instructor-led group training plus two to three hours of self-study. Compared to performance at pretest and performance of inactive waitlist control participants, training produced improved name recall memory, higher levels of memory self-efficacy, and more effective use of memory strategies. Critically important, the benefits from EMC-R training extended beyond the targeted name recall task that was trained to perform on similar, but untrained, associative memory tasks. Looking at the power of self-regulatory factors, there was no additional benefit from the SB approach, which added more self-regulatory elements to the training program. At the same time, significant training-related performance gains were mediated by self-regulatory gains in strategy usage and memory self-efficacy.

### 4.1. Effectiveness of Brief Memory Training Program

The first aim of this EMC-R study was to test the effectiveness of an abbreviated memory strategy training program focused on techniques for name recall. Consistent with previous research [12,20], the training program was effective in terms of performance gains on the target task. The primary contribution to the cognitive intervention literature here is that EMC-R was also effective in enhancing memory self-efficacy and effective strategy use, which have rarely been directly assessed in previous research. By enhancing self-regulatory factors, EMC-R showed increased “bang for the buck,” in terms of benefits beyond improved name recall, which are important to practitioners and trainees alike.

Most training programs have not directly assessed gains in self-evaluative beliefs for middle-aged and older adults, but among programs that did, only some were successful [16]. A meta-analysis [65] suggested that memory training can improve subjective beliefs about memory, although this effect was of much smaller magnitude than the benefit to memory performance. Importantly, EMC-R was unique from the past successful training programs because of its shorter duration and lower intensity. The original EMC project [26], successful in enhancing trainees’ control beliefs and memory self-efficacy, lasted five weeks. ACTIVE trainees demonstrated improved control beliefs after ten weeks of training, but only for speed and reasoning training, not for memory training [66]. Other investigators reported changes in memory self-efficacy or control beliefs after 16 [16] or 12 h of training [67]. Clearly, the duration of these training programs far exceeded that of EMC-R, and there has been limited evidence of the benefits to beliefs from short-term training. In fact, Woolverton and colleagues [68] directly compared “short” and “long” versions of memory training. The short version (13 h) was less effective. Thus, the self-evaluative belief gains in EMC-R are remarkable given that this training was comparatively short in relation to other successful programs. It may be that the homework requirement, while reducing in-group training time, also gave participants the opportunity to see that they could work with the strategies on their own, which may have led to more positive beliefs.

In addition to enhanced self-evaluative beliefs, EMC-R trainees reported more effective strategy use than control participants. Evidence of effective strategy use by EMC-R trainees is important because change in strategy use is often assumed but rarely assessed [16]. A review of strategy use across twelve interventions suggested that trainees increased strategy use over time, compared to control participants [20], but more research is needed to establish the extent to which strategy use changes as a function of training. For example, few EMC trainees reported using all the trained strategies, and many reported only using the simpler parts of advanced strategies [26]. This finding is consistent with research that suggests older adults may over-rely on familiar strategies [31].

The use of more strategies represents self-regulation in that it requires flexible adaptation to the task at hand, in response to continued monitoring of performance. This self-regulation via effective strategy use was indicated by EMC-R trainees: EMC-R trainees reported using a greater number of strategies overall, and a greater proportion of trainees, compared to control participants, reported preferred use of associative strategies that were directly targeted in training, and were related to superior performance. At the same time, few trainees made the extensive effort to learn and utilize the complicated image–name match method taught in class. This suggests that trainees were selectively focusing their strategic effort on techniques that were learned in training and were advantageous while still being relatively easy to employ, both in class and while doing the homework assignments.

These benefits of training are notable, but training can only be effective if it can be completed. The true strength of EMC-R is the possibility of broad dissemination, given its brief duration. Training that occurs over a single week, with only one 2-h class plus homework, could easily be offered at lifelong learning programs. Future work might assess whether EMC-R could successfully be implemented by activities staff or volunteer peer leaders, with minimal training [69].

Additionally, scholars suggest that training should focus on specific memory tasks that are important to trainees [31,68], and some of EMC-R’s success may have been derived from the high value that middle-aged and older adults place on name recall [1,51]. Thus, replications of this brief training focused on other types of everyday memory, such as story recall, which was improved in EMC [26], would logically extend the impact of this training.

### 4.2. Evidence for Near Transfer

Evidence of transfer is a central contribution of the EMC-R project—even though the EMC-R strategies and practice exercises focused on learning and remembering name–face pairs, trainees, but not control participants—demonstrated improved performance on occupation–name recall tasks. These represented near transfer, as they showed higher correlations with name recall than the other transfer tasks. Evidence for near transfer is particularly important given the controversy surrounding generalization and transfer in cognitive interventions [31] and the decidedly limited past evidence for transfer after training focused on strategy learning.

Specific elements of EMC-R’s design may have contributed to the successful evidence of near transfer. Barnett and Ceci [70] proposed that transfer is most likely (1) when transferring from a “deep” general principle to a “superficial” specific task, (2) when the trained and transfer outcomes have similar requirements, and (3) when the successful implementation of the trained procedures to the transfer task requires minimal memory demand. EMC-R met these criteria. First, the training program was multifactorial. Trainees learned “deep” general principles—the importance of attention and how to enhance it, how memory works, and expected age-related memory changes—and then covered multiple specific strategies. Second, both the target and the transfer assessments, as well as the practice exercises, focused primarily on accuracy as the outcome, rather than speed. Taken a step further, both the target task and the transfer tasks required associative memory skill, which relies on the ability to bind two pieces of information together, a skill which often declines in late life [71], but which was emphasized in training. Further, the tasks that showed transfer were those that were most similar to the trained name recall task. Even though there was some challenge involved—participants would have needed to recall the strategy that they learned while evaluating its effectiveness for the new task at hand—it was less demanding than trying to apply the techniques to a completely different type of task. In fact, the more dissimilar object location task did not show transfer.

Other factors may also have contributed to the transfer here. The low attrition rate in this study, which was unrelated to the experimental condition, suggested that participants may have had high intrinsic motivation to maximize training benefits, which may have promoted transfer [24]. Learning more strategies may also have made it possible to adapt known techniques to new associative memory tasks. Bandura [44] also posited that self-efficacy is key to task-motivated behavior, task persistence, and, ultimately, task success. Reasonably, if an EMC-R trainee felt more confident in their ability to complete memory activities, they might maintain effort longer on the challenging occupation–name task, and the addition of homework assignments may have aided in supporting confidence as noted earlier. Similarly, the flexible adaptation and performance monitoring that likely underlies effective strategy use might easily translate to tasks requiring a similar associative approach.

### 4.3. The Value of Self-Regulation in Training Approaches

The previous EMC project, on which this research was based, applied self-efficacy theory [44] in its design and was successful in enhancing trainees’ self-regulation, as well as memory performance [26]. Because EMC research compared trainees to inactive control participants, the specific impact of this emphasis on self-regulation in the training design was unknown. In EMC-R, we were able to examine the importance of self-regulation by testing the differential impact of the added self-regulatory elements for the SB group, and by examining the extent to which the observed self-regulatory gains in training mediated memory gains.

Considering the modest evidence for self-regulatory benefits from relatively long training programs (discussed above), it may be that training gains have derived simply from completing memory strategy training, and not from a training approach focused on self-regulation. The present research aimed to clarify this issue by explicitly providing self-regulatory elements in two different training conditions (modeling, group support, extensive practice) and assessing the benefit of additional self-regulatory components in one of those conditions. No relative benefit for the beliefs-focused SB group was found, in terms of gains in memory performance or self-regulation.

Potentially, training differences between SB and SO were comparable due to the brief time course of training and testing. Additional time in the training itself may be needed to reinforce the self-regulatory components. Trainees might also require additional time following training to process training lessons in order to maximize the potential of newly-learning strategies. In EMC, self-evaluative beliefs were assessed one month, but not one week, following training [26], and latent growth curve modeling suggested that self-evaluative beliefs and training-related gains in these beliefs influenced memory performance one month following training [22]. A future project could replicate the EMC-R name recall training with follow-up assessments at least one month after training.

In theory, better self-regulation might enhance training effects to the extent that it promotes persistent effort when facing challenges. Past research shows that compliant EMC trainees who were more engaged with the training program, and completed more of the activities, gained more from training [72]. Further, in a working memory training paradigm, greater motivation as operationalized by a greater need for cognition or a growth mindset was related to more training-related gains [24]. In this particular study, the relatively high intrinsic motivation across both training groups, or other individual characteristics of trainees, might have “overridden” the possible gradation of gains between the SB and SO training approaches. This supposition could be tested by assessing motivational factors prior to training, pre-selecting individuals with lower scores on self-regulation measures, or by manipulating motivation via varied participant recruitment or payment procedures [24].

Coupled with the presence of factors that boosted self-regulation in both groups, the most parsimonious explanation for the comparable training outcomes for SB and SO was the limited training duration, which might be viewed as a reduced “dose” of training. Although several differences between the two training approaches were documented (see Methods), the abbreviated nature of EMC-R may not have allowed for sufficient emphasis on the extra SB elements that could have enhanced self-regulation. Some elements to enhance self-regulation are present in most group strategy training, such as the repeated practice of skills and the presence of social support from other group members. Indeed, this may be why group training is consistently more effective in terms of memory gains than individual, self-help training [8]. SB provided more support and encouragement from the instructor than SO, but it may not matter who provides positive feedback or emphasizes the value of using trained strategies. To better understand the role of groups in beliefs-focused training, future work might use self-study individual training and active social non-memory groups as control conditions.

There are other possible elements to enhance self-regulation that were limited here or were impractical to include within a single week. For instance, the longer EMC training program also included greater opportunities for in-class success using a variety of strategies, additional readings, and small-group discussions to help trainees understand the lessons. The most important of the EMC elements, dropped here due to time constraints, may have been explicit goal setting for performance gains and positively framed objective feedback on the trainees’ progress after a mid-course evaluation. Goal setting and feedback may be critical for boosting training gains, given their impact on performance across adulthood [73,74,75,76]. Comparison of training approaches with and without goal setting and provision of objective feedback on interim training gains are recommended if investigators can identify a way to incorporate goal setting and feedback while still maintaining a relatively short-term training approach.

In addition to examining the relative benefits of SO and SB training, to enhance our understanding of the relationship between memory training impact and self-regulation, we also explicitly tested the mediation of training gains by self-regulatory change. Critically, we found that the training-related gains from before training to after training (pretest-posttest) were fully mediated by change in memory self-efficacy and change in reported strategy use. Both indirect paths—through memory self-efficacy and strategy use—were significant and contrast testing suggested they were of equal value in explaining memory performance. Compared to waitlist control participants, trainees felt more confident in their memory ability and reported using more strategies on the memory test over time. In turn, the increased confidence and greater strategy use are what explained the observed training-related benefits to name recall performance. This is a very important finding. First, we established that brief training may lead to improvements in self-regulation. Further, these gains were critical predictive mechanisms for the observed benefits of training on targeted abilities.

### 4.4. Limitations

While the results of this research are quite promising, some possible limitations could be addressed in future research, including study recruitment and sampling issues. The majority of the study sample was female (see Appendix C, Table A2). Well-documented sex differences evidence that women outperform men on episodic recall, name recall, and face recognition in mid-life and old age [77,78]. Importantly, because the proportion of female participants was comparable across the three experimental conditions, these sex differences were likely not a confound here. The participants were also relatively well-educated and healthy. Some scholars propose a Matthew Effect, wherein those persons who are advantaged in regards to cognitive capacity, education, or health, benefit most from training. Alternatively, the greatest gains might be evidenced by those who have more to gain [9,34]. Future research is needed to determine whether men, or less healthy or less well-educated individuals would benefit as much or more from this training approach.

Participant recruitment materials advertised an everyday memory training program, and participant payments were the training materials. Thus, participants may have self-selected based on interest in improving memory skills. Considering this, the study sample may have had greater subjective complaints or more concerns about memory than the general population. Such selectivity could be concerning because subjective memory complaints are related to objective memory performance and may be a precursor to cognitive decline [6,79] However, pretest memory self-efficacy and general memory evaluation scores for this study were consistent with baseline values observed across six non-training memory studies from the same geographic area in Florida [55,56,80,81,82]. These data suggest that the sample was not disadvantaged in terms of memory complaints. Further, because participants in the three different training conditions did not differ in baseline measures of working memory, episodic memory, or general memory evaluation, motivational factors were unlikely to have confounded comparisons between groups.

Another potential limitation has to do with the EMC-R time frame. Cognitive researchers and aging persons alike are concerned with the temporal transfer of training and question whether training-related gains are sustained, and, if so, for how long [9]. Evidence for long-term training benefits is limited [83,84]. Training-related gains from the present study may not have persisted beyond the week, or they might have been sustained or even magnified, and the long-term trajectory of training effects may differ between the SB and SO conditions. Furthermore, due to the importance of validating a training format that was brief and easily replicable in community settings, it was not feasible to examine specific brain mechanisms related to the behavioral changes we observed. These possibilities should be tested in future work with long-term follow-up assessments.

## 5. Conclusions

Collective results from cognitive intervention research demonstrate that middle-aged and older persons may show test score improvements with small amounts of training [8,9], but researchers and trainees alike are concerned with the true practical impact of these programs. Researchers debate whether training can modify self-regulation—how people evaluate their abilities and approach memory tasks—as this could promote self-sustaining performance gains [11,23]. Controversy also surrounds the scope of training benefits: are gains limited to performance on the targeted tasks? EMC-R provides insight towards the resolution of these controversies.

When considering interventions for memory and aging, it might be tempting to focus on improved memory performance per se. EMC-R did significantly improve memory on both trained and untrained tasks. However, performance is often determined not solely by ability level, but rather by the success of a comprehensive self-regulatory system including personal, behavioral, and environmental factors [44]. As such, the true importance of EMC-R was that the training boosted specific self-regulatory factors, namely memory self-efficacy and effective strategy use. These factors were key to memory success at posttest and enhanced self-regulation may be the “special sauce” necessary for near transfer [11,24,25]. Evidence of near transfer to other cognitive tasks has been limited [11]. Thus, the modest evidence of near transfer in EMC-R is remarkable. The direct value of strategy training for middle-aged or older trainees is heightened if the benefits of the program are not limited to the specifically trained task.

Collectively, EMC-R underscores the value of brief strategy training focused on self-regulation. Five hours over a single week (class time plus homework) enhanced performance on the trained memory task, but also improved self-regulation in terms of strategies and beliefs, and performance on another associative task that was neither mentioned nor practiced in training. From a practical standpoint, this successful brief format for training could thus be easily incorporated into existing programming in a Senior Center or lifelong learning program. Further, the low cost of a single session program—in terms of both trainer time and participant time—may incentivize initial participation and full completion of training, leading to changes that can potentially contribute to daily memory function and independent living.

## Figures and Tables

**Table 1:** Week-by-week overview of assessment and training procedure.

Week	Groups	Agenda
0	All	Phone interview: Eligibility, demographics, and other control measures
1	All	Pretest assessment: Primary and transfer outcomes
2	SB	2-h group training session, followed by ~2 h of self-study in workbook
	SO	2-h group training session, followed by ~2 h of self-study in workbook
	Control	No meeting, homework, or other activity
3	All	Posttest assessment: Primary and transfer outcomes

Note. “All” includes both training groups and waitlist control.

**Table 2:** Overview of measures with references.

Measure
***Phone Interview***
Verbal Informed Consent
Telephone Interview for Cognitive Status (TICS) [52]
Rey Auditory-Verbal Learning Test (RAVLT) [53]
Backward Digit Span from WAIS-III [53]
Short Form Health Survey (SF-12) [48]
Perceived Mastery [54]
General Memory Evaluation (GME) [55]
Surveys of medication/supplement use, ratings of sensory function, demographic information
***Targeted Pretest/Posttest Assessments***
Written Informed Consent *
Name Recall (Levels 1 and 2) [26]
Memory Self-Efficacy Questionnaire (MSEQ-4) [56]
Locus of Control for Memory (Metamemory in Adulthood Questionnaire subscale) [57]
Occupation–Name Verbal Association [58,59]
Object–Location Visual Association [60,61]
Strategy Use Checklists (for name recall, occupation–name association, and object–location association) [26]
***Exploratory Assessments***
Memory Anxiety (Metamemory in Adulthood Questionnaire Subscale) [57]
Mindful Attention Awareness Scale (MAAS) [62]
Subjective Age Identity [51]
Surveys of health and memory engagement

* Administered at Pretest only.

**Table 3:** List of training elements to enhance self-regulation included in each group.

Self-Efficacy Elements	Control	SO	SB
***Enactive mastery***			
Each skill practiced repeatedly	-	✓	✓
In class success remembering names of class members *	-	✓	✓
Success with strategy practice	-	✓	✓
Easier strategies first	-	-	✓
***Vicarious experience***			
Trainer gives examples, models each strategy	-	✓	✓
Emphasis on learning from each other in class	-	-	✓
Whole group practices strategy together	-	✓	✓
***Verbal persuasion***			
Group provides social support *	-	✓	✓
Positive feedback from trainer in session	-	-	✓
Readings emphasize potential at any age	-	-	✓
Research presented on strategy effectiveness and learning potential of seniors	-	-	✓
Trainer reframes all negative comments to emphasize potential	-	-	✓
***Physiologic and affective states (reducing anxiety*)**			
At home training materials to allow self-pacing or sufficient time for learning	-	✓	✓
Readings reviewed in class to help trainees understand	-	✓	✓
Class discussion shows that others have similar problems	-	✓	✓
Emphasis on process/learning, not on 100% score	-	-	✓
Focus on potential in readings and in class sessions	-	-	✓
Emphasize personal decision-making about what to learn	-	-	✓
Trainer emphasizes that everyone has memory failures	-	-	✓

* While this element may occur naturally in group training, the train emphasized or encouraged in the SB group, but not in the SO group.

**Table 4:** Observed means and standard deviations for primary outcomes for trainees and control group at pretest and posttest.

	Control (*n* = 38)		Trained (*n* = 79)		Total (*N* = 117)	
	*M*	*SD*	*M*	*SD*	*M*	*SD*
Name recall (% correct)						
Pretest	47.37	22.52	46.52	25.34	46.79	24.37
Posttest	47.04	23.29	53.85	24.53	51.64	24.25
Memory self-efficacy (0–100)						
Pretest	41.96	16.45	39.11	16.95	40.03	16.77
Posttest	44.79	16.08	48.09	16.58	47.02	16.42
Memory control (1–5)						
Pretest	3.42	0.54	3.45	0.50	3.44	0.51
Posttest	3.47	0.47	3.62	0.52	3.57	0.51
Name recall strategy use (% used)						
Pretest	26.78	10.39	26.14	9.87	26.34	10.00
Posttest	29.88	12.26	34.55	11.55	33.03	11.94

**Table 5:** Observed means and standard deviations for primary outcomes for SO and SB trainees at pretest and posttest.

	SO (*n* = 44)		SB (*n* = 35)		Total (*N* = 79)	
	*M*	*SD*	*M*	*SD*	*M*	*SD*
Name recall (% correct)						
Pretest	50.19	25.59	41.90	24.61	46.52	25.34
Posttest	57.20	23.59	49.64	25.37	53.85	24.53
Memory self-efficacy (0–100)						
Pretest	40.19	16.85	37.74	17.22	39.11	16.95
Posttest	50.31	15.85	45.31	17.27	48.09	16.58
Memory control (1–5)						
Pretest	3.51	0.47	3.38	0.54	3.45	0.50
Posttest	3.63	0.56	3.60	0.49	3.62	0.52
Name recall strategy use (% used)						
Pretest	25.27	8.38	27.23	11.51	26.14	9.87
Posttest	35.03	10.98	33.95	12.36	34.55	11.55

**Table 6:** Observed means and standard deviations for transfer outcomes by training condition at pretest and posttest.

	CT (*n* = 38)		SO (*n* = 44)		SB (*n* = 36)		Total (*N* = 118)	
	*M*	*SD*	*M*	*SD*	*M*	*SD*	*M*	*SD*
Occupation-name immediate recall (%)								
Pretest	28.86	16.93	30.00	21.39	22.04	19.86	27.20	19.72
Posttest	30.00	19.25	36.21	20.84	30.46	22.14	32.46	20.78
Occupation-name delayed recall (%)								
Pretest	25.00	15.02	26.06	21.74	18.61	18.91	23.45	19.04
Posttest	26.93	17.85	31.89	20.99	28.15	22.29	29.15	20.40
Occupation-name delayed recognition (%)								
Pretest	53.51	22.15	54.09	24.45	41.30	25.98	50.00	24.71
Posttest	57.46	21.87	59.92	21.51	53.89	24.06	57.29	22.38
Object-location delayed recall (%)								
Pretest	62.99	20.36	66.70	24.34	56.55	24.39	62.40	23.40
Posttest	63.76	19.93	64.73	21.27	58.57	25.05	62.61	22.09

## Data Availability

Upon publication, data will be made available in a publicly accessible repository.

## Supplementary Materials

The following supporting information can be downloaded at: https://www.mdpi.com/article/10.3390/brainsci12040465/s1. Supplementary Material A: Additional details on baseline, control, and exploratory measures; Supplementary Material B: Name-face test stimuli examples; Supplementary Material C: Strategy checklists; Supplementary Material D: Object-location association material; Supplementary Material E: Name-occupation association materials; Supplementary Material F: Training homework instructions.

## Appendix A. Participants: Participant Exclusion, Attrition, and Demographics

Twenty-one individuals completed the phone interview but were ineligible for the study due to performance on the Telephone Interview for Cognitive Status (TICS) [52], scoring below the recommended cut-off score of 31, which was suggestive of cognitive impairment. An additional 23 individuals completed the telephone interview but did not participate in any other part of the research program, due to scheduling issues (*n* = 10), illness (*n* = 9), death in the family (*n* = 2), and unspecified reasons (*n* = 2).

One participant did not complete the first in-person assessment due to scheduling issues. Four participants did not complete the second in-person assessment, withdrawing after completion of training due to unspecified reasons (*n* = 2), health issues (*n* = 1), and lack of interest (*n* = 1). Two of these participants were assigned to the strategy-only training group, and the other two were assigned to the strategy-plus-beliefs training group, suggesting that the attrition was not due to condition assignment.

Participants who dropped out of the study after the pretest and training session were similar to those who completed the posttest interview. Independent sample *t*-tests compared mean scores of these two groups for age, self-rated vision, hearing, physical health and mental health, years of education, number of prescription medications taken regularly, baseline cognitive performance (Telephone Interview for Cognitive Status, Backward Digit Span, RAVLT immediate and delayed list recall), and all primary outcomes and transfer outcomes assessed at pretest. Levene’s test was not significant (all *p*’s > 0.05) for the comparison of any of these measures across either group, suggesting that the assumption of homogeneity of variance was tenable. Mean scores for participants who dropped out of the study following the pretest interview were comparable to those for participants who completed the posttest interview, except that those who dropped out had lower scores on the Telephone Interview for Cognitive Status (*M*_diff_ = −2.44, *SE* = 1.06), *t*(120) = −2.30, *p* = 0.023, rated their correct vision as poorer (*M*_diff_ = −1.54, *SE* = 0.67), *t*(119) = −2.27, *p* = 0.025, and reported using fewer strategies on the face–name association test (*M*_diff_ = −10.17, *SE* = 5.06), *t*(119) = −2.01, *p* = 0.047, suggesting that ability factors may have influenced this attrition.

Participants reported taking an average of 2.66 prescription medications regularly. About one-third (30.8%) of participants reported taking one or fewer prescription medications regularly and only 8.3% reported taking five or more prescription medications regularly. Only 16 participants (13.3%) reported being hospitalized in the previous year.

## Appendix B. Training Procedures: Examples to Compare Self-Regulatory Elements in Training Groups

**Table A1:** Examples to compare written self-regulatory elements in two training groups.

SB Version	SO Version
1. Workbook Introductory Paragraph	1. Workbook Introductory Paragraph
We recognize that each person begins on a different level. We all start with different skills and different ability levels. Each person has different memory experiences. By beginning with fairly simple techniques, we hope to ensure that all of you understand and benefit from this lesson, even if you have very little background in memory. The Everyday Memory Training program is not about being an expert memorizer. It’s not about getting 100%. It’s about each one of you learning and improving, so that you will be able to remember more after the program is done.	Memory is complicated. We have been working on memory research for many years. So we are going to try to teach you specific techniques that might help you to improve your memory performance. Because there are many different techniques, you will learn more than one technique in class. Some of these techniques will be hard and other memory methods will be easy. And you will be asked to do practice exercises with these techniques. The lesson in class today and the readings and practice exercises are intended to help you to raise your memory scores.
2. Workbook Closing Paragraph	2. Workbook Closing Paragraph
In this memory training program, you have learned how to use different memory techniques. We have discussed association, imagery, the image-name-match method and active observation. Research evidence tells us that each of these techniques can support your memory in general and make it more likely that you will remember names. The homework section of this notebook starts on the next page. The homework includes readings and memory activities that will give you an opportunity to practice and master the memory techniques that you learned today.	In this memory training program, you have learned how to use different memory techniques. We have discussed association, imagery, the image-name-match method and active observation. Each of these techniques can support your memory in general and make it possible to remember names. The homework section of this notebook starts on the next page. The homework includes readings and memory activities that will improve your memory scores.
3. Excerpt from General Instructions for Practice Exercises	3. Excerpt from General Instructions for Practice Exercises
You have an activity log with this packet so that you can keep track of your exercise time. The more you practice, the better you will do on these tasks. It is up to you. With additional practice, you are more likely to master the strategies you learned in class.	You have an activity log with this packet so that you can keep track of your exercise time. The more you practice, the better you will do on these tasks.

## Appendix C. Results: Baseline Statistics by Training Condition

**Table A2:** Baseline statistics by training condition.

	CT (*n* = 38) 79% Female 95% White		SO (*n* = 46) 76% Female 89% White		SB (*n =* 38) 82% FEMALE 92% white		Total (*N* = 122) 79% Female 92% White	
	*M*	*SD*	*M*	*SD*	*M*	*SD*	*M*	*SD*
Age (years) ^a^	72.51	8.34	71.51	7.34	76.08	8.83	73.24	8.31
Years of education	17.76	3.07	17.50	2.96	16.68	2.36	17.33	2.84
Rating of English ability (1–10)	9.32	0.93	8.91	1.31	9.03	0.97	9.07	1.11
Rating of corrected vision (1–10)	8.08	1.44	8.17	1.06	7.68	1.56	7.99	1.38
Rating of corrected hearing (1–10)	7.87	1.55	8.33	1.52	7.97	1.64	8.07	1.57
RAVLT immediate recall (0–15)	7.68	2.41	7.28	2.23	7.21	2.71	7.39	2.42
RAVLT delayed recall (0–15)	4.11	2.47	4.37	2.41	4.34	2.91	4.28	2.60
Backward digit span (2–8)	5.13	1.42	5.26	1.34	5.11	1.20	5.17	1.31
Physical health (PCS from SF-12)	50.25	8.79	49.05	10.14	47.81	8.99	49.04	9.36
Mental health (MCS from SF-12)	54.67	7.12	54.39	6.60	55.03	6.13	54.68	6.58
Perceived mastery (1–6)	5.19	0.65	5.00	0.77	4.99	0.91	5.06	0.78
General memory evaluation (1–7)	4.59	1.24	4.18	1.22	4.04	1.24	4.26	1.24

^a^ Significant condition difference, *p* < 0.05; CT = Waitlist control participants. SB = Strategy-plus-beliefs training group. SO = Strategy-only training group.

## Appendix D. Results: Correlations for Pretest and Posttest Name Recall and Transfer Outcomes

**Table A3:** Correlations for pretest and posttest name recall and transfer outcomes.

Measure	1	2	3	4	5
1. Name recall (level 2)	–	0.60	0.63	0.51	0.46
2. Occupation–name immediate recall	0.60	–	0.96	0.74	0.45
3. Occupation–name delayed recall	0.62	0.97	–	0.75	0.46
4. Occupation–name delayed recognition	0.47	0.71	0.71	–	0.52
5. Object–location delayed recall	0.47	0.41	0.45	0.34	–

Note. All coefficients are significant at *p* < 0.001. Intercorrelations for pretest are presented above the diagonal, and intercorrelations for posttest are presented below the diagonal. Each correlation was conducted using the maximum number of participants who had complete data for both measures (pretest *n* = 120–122; posttest *n* = 114–118).
