# Supplementary material for: Brief Strategy Training in Aging: Near Transfer Effects and Mediation of Gains by Improved Self-Regulation
Source: Brain Sci. 2022 Mar 30;12(4):465. doi: 10.3390/brainsci12040465 (PMC9027409; doi:10.3390/brainsci12040465)
Supplement: Supplementary file 1 [file brainsci-12-00465-s001.zip › brainsci-1641866-supplementary.pdf]

# Supplemental Materials

## Contents

Supplementary Materials A. Measures and Materials: Baseline, Control, and Exploratory Measures..........2

Supplementary Materials B. Primary Outcome Measures: Name-Face Test Stimuli.......................................5

Supplementary Materials C. Primary Outcome Measures: Strategy Checklists...............................................6

Supplementary Materials D. Transfer Outcome Measures: Object-Location Association Materials..............9

Supplementary Materials E. Transfer Outcome Measures: Name-Occupation Association Materials........12

Supplementary Materials F. Training Procedures: Homework Instructions ..................................................13

### Supplementary Materials A. Measures and Materials: Baseline, Control, and Exploratory Measures

*SM.1. Background and Control Measures*

Background and control measures were assessed during the phone interview. Background measures included participants' responses to surveys on medication and supplement use, demographic information (e.g., years of education, gender, date of birth) and ratings of English language ability and sensory function. Additional measures included a screening for cognitive function (Telephone Interview for Cognitive Status; TICS), a test of episodic memory (Rey Auditory-Verbal Learning Test; RAVLT), a test of working memory (Backward Digit Span), and surveys of self-reported health (SF-12), general personal control beliefs (Perceived Mastery), and general beliefs about ones' memory ability (General Memory Evaluation; GME). Each of these measures are known to be reliable and valid if administered over the phone to older adult populations.

SM.1.1. Telephone Interview for Cognitive Status (TICS)

The Telephone Interview for Cognitive Status (Brandt et al.,1988) is a telephone screening for cognitive impairment designed for older adults. The TICS includes 11 questions, with a maximum score of 41 points. Higher scores indicate better cognitive performance. Example items include asking participants to identify the complete date, their current location, and the first and last names of the American president and vice president. Participants are also asked to count backwards by sevens from 100 five times, to provide antonyms for common words, name specific items, repeat phrases, and tap on the phone five times. The TICS is highly sensitive and specific, is strongly correlated to performance on the widely-used Mini-Mental State Examination, $r = .94$, and has strong test-retest reliability, $r = .97$. Following the guidelines tested by Brandt et al. (1988), participants eligible for the present research scored greater than 30 out of 41, suggesting freedom from cognitive impairment.

SM.1.2. Rey Auditory-Verbal Learning Test (RAVLT)

The Rey Auditory-Verbal Learning Test (Lachman, Agrigoroaei, Tun, & Weaver, 2014; Lezak, 1995) assesses episodic verbal memory. RAVLT includes immediate and delayed free recall of a list of 15 commonplace nouns, such as *flower* and *school*. During encoding, research assistants read the 15 words, with approximately 1-second pause between each word. Participants were given 90 seconds immediately following encoding to recall as many of the words as they could remember. Following an approximate ten-minute delay, participants were given 60 seconds to recall the words again. Scores were calculated as the

number of words correctly recalled, ranging from 0 to 15. Higher scores represent better episodic memory performance. Number of intrusions provided were also counted.

SM.1.3. Backward Digit Span Test

The Backward Digit Span test, adapted from the WAIS-III for use in the Brief Test of Adult Cognition by Telephone (Lachman et al., 2014; Wechsler, 1997), assesses working memory span. Research assistants read strings of digits at a rate of one second per digit. Participants were asked to repeat the digits in the reverse order. Digit span increased from a length of two digits to a length of eight digits. If participants missed the first string at one level (length of string), a second item at that level was administered. The task was discontinued if a participant incorrectly answered both items at a single level. The backward digit span score was calculated as the longest digit span correctly repeated backwards at least once, with a maximum score of 8. Higher scores represent greater working memory capacity.

SM.1.4. Short Form Health Survey (SF-12)

Self-reported physical health and mental health were assessed using the Short Form Health Survey (SF-12; Ware, Kosinski, & Keller, 1996). The SF-12 includes twelve items that use various response scales, ranging from dichotomous response options of Yes / No to 6-point Likert scales. One item assesses overall general health (In general, would you say your health is, with responses from 1 = excellent to 5 = poor), and the other items assess the extent to which persons are limited as a result of physical or emotional problems (e.g., During the past four weeks, how much did pain interfere with your normal work, including both work outside the home and housework?, with 5-point Likert scale response options from Not at all to Extremely). Summary scores for the physical component scale (PCS) and the mental component scale (MCS) were calculated following procedures outlined by Ware and colleagues (1995). These norm-based standardized scores are continuous and calculated to have a mean of 50 and a standard deviation of 10 in the U.S. adult population. Higher values represent better physical or mental health. The internal consistency of the SF-12 was adequate (Cronbach's $\alpha$ = .81), consistent with previous research (Strickland-Hughes et al., 2016) although the items were selected to represent several dimensions of physical and mental health and are thus heterogeneous. The SF-12 has good relative validity when compared to a longer form of the health survey and other health criteria and measures (Ware et al., 1996).

SM.1.5. Perceived Mastery Survey

The Perceived Mastery scale (Lachman & Weaver, 1998) assesses respondents' general sense of personal control. This provided a baseline score representing a global analog to the domain-specific personal locus of control for memory of interest in this research. The present research used the 5-item version of the Perceived Mastery scale adapted from Pearlin and Schooler (1978) by Lachman and Weaver (1998) and used in the Health and Retirement Study (HRS; http://hrsonline.isr.umich.edu; Hauser & Willis, 2005), a large-scale longitudinal study. Example items include: When I really want to do something, I usually find a way to succeed at it and What happens to me in the future mostly depends on me. Responses were made using a 6-point Likert scale ranging from 1 = strongly disagree to 6 = strongly agree. A perceived mastery score was computed as the mean of the responses to these five items (range: 1 – 6). Higher scores represent greater sense of perceived mastery. The scale had adequate internal consistency reliability, Cronbach's $\alpha$ = .78, although this reliability was not as strong as that reported by the 2006, 2008, and 2010 waves of HRS (Cronbach's $\alpha$ = .89 - .90). Internal consistency would not have been higher if any of the items were removed.

SM.1.6. General Memory Evaluation Survey (GME)

The General Memory Evaluation survey (West, Dark-Freudeman, & Bagwell, 2009) assesses global beliefs about memory, comparable to the specific memory self-efficacy measure of interest in this study,

and thus represents a baseline score for memory self-efficacy. The three items in the GME concern evaluation of recent memory performance, comparison of one's memory to same-aged peers, and overall satisfaction with recent memory performance. An example item is How satisfied are you with your recent memory performance? Participants rate three items using a 7-point Likert scale (e.g., 1 = very unsatisfied to 7 = very satisfied). An index GME score was calculated by averaging responses to the three items (range: 1 to 7). Higher scores indicate greater perceived general memory ability. This score had good internal consistency reliability, Cronbach's $\alpha$ = .84, consistent with previous research (Strickland-Hughes et al., 2016).

*SM.2. Exploratory Measures*

The following exploratory measures were administered at the pretest and posttest assessments. These measures were selected because research suggests that they are related to memory performance and/or self-regulation and may benefit from training or moderate training effects, but they were not central to any of the study aims or hypotheses.

SM.2.1. Memory Anxiety

Memory anxiety, or perceptions of the relationship between anxiety and memory performance, was be assessed with the 14-item Anxiety subscale of the MIA (Dixon et al., 1988). Example items include I find it harder to remember things when I'm upset and I feel jittery if I have to introduce someone I just met. As with the Locus subscale of the MIA, item responses were made using a 5-point Likert scale, and an index score was calculated by averaging all responses. A higher score (range: 1 – 5) indicated greater memory-related anxiety. The Anxiety subscale had good internal consistency reliability (pretest Cronbach's $\alpha$ = .87, posttest Cronbach's $\alpha$ = .89), consistent with previous literature (Cronbach's $\alpha$ = .83 - .87 across four studies; Dixon et al., 1988).

SM.2.2. Mindful Attention Awareness Scale (MAAS)

The State Mindfulness subscale of the Mindful Attention Awareness Scale (Brown & Ryan, 2003) represents the extent to which individuals pay attention to the present moment. Mindful attention may benefit from the strategy training to the extent that name recall strategies aim to focus attention. Participants rated five items using a 7-point Likert scale, anchored at 0 = not at all, 3 = somewhat, and 6 = very much. Example items include I find myself doing things without paying attention and I find it difficult to stay focused on what's happening in the present. An index score was calculated by averaging the five responses (range: 0 – 6). Higher scores will represent greater attentiveness. MAAS had acceptable internal consistency reliability (retest Cronbach's $\alpha$ = .85, posttest Cronbach's $\alpha$ = .84), although somewhat lower than previous research with a larger sample across adulthood (Cronbach's = .92; Brown & Ryan, 2003).

Subjective Age Identity. Subjective age reflects how old individuals feel. Subjective age may be important for the present research, given the preponderance of negative aging stereotypes about memory and their effects on performance (Chasteen, Kang, & Remedios, 2011; Hummert, 2011). Participants responded to five subjective age identity questions that were adapted from previous research (Kastenbaum, Derbin, Sabatini, & Artt, 1972) and administered in Strickland-Hughes et al. (2016) by indicating the ages that they feel. They reported their felt age (At this moment, how old do you feel?) and desired age (If you could choose your age, how old would you want to be?), among others. Proportional subjective age identity scores were calculated using the average of each individual's five subjective age responses, specifically: (Subjective Age – Chronological Age) / Chronological Age $\times$ 100. Proportional subjective age identity scores represent the percentage of one's life that he or she feels younger or older than his or her chronological age. For example, if a woman aged 75 years old reported an average subjective age of 50 years, her proportional age identity score would be -33.33, indicating that she feels 33% younger

than she is. This measure had strong test-retest reliability, r = .97, and internal consistency reliability, Cronbach's $\alpha$ = .97, in past research (Strickland-Hughes et al., 2016), and adequate internal consistency reliability in the present research (pretest Cronbach's $\alpha$ = .82; posttest Cronbach's $\alpha$ = .82).

SM.2.3. Health and Memory Engagement Information

A survey assessing health and memory-engagement non-ability factors that might affect memory performance was administered. This survey was created for the present research and is available upon request. The health-related items, such as whether the participant has been hospitalized recently and a list of current medications taken, were collected for post hoc examination in case participants had difficultly following instructions or seemed confused. Memory engagement items included four frequency ratings of memory activities (e.g., exercise my memory with games or activities not on a computer, a = *never* to i = *daily*) and reports of participation in other formal memory training programs. An index score was calculated by averaging responses to the four frequency ratings (range: 1 – 9), with higher scores indicating a greater frequency of engagement in memory-related activities. This scale had weak internal reliability consistency (pretest Cronbach's $\alpha$ = .64; posttest Cronbach's $\alpha$ = .71), which was unsurprising given that each of the items assessed the frequency of discrete memory-related activities.

**Supplementary Materials B. Primary Outcome Measures: Name-Face Test Stimuli**

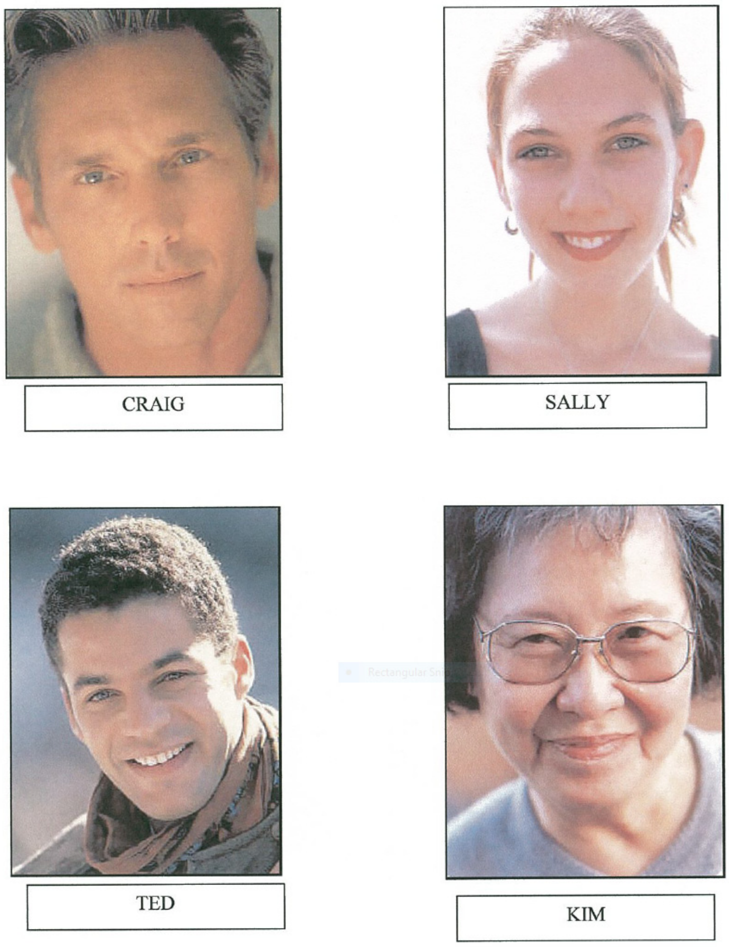


**Figure S1.** Example of page of to-be-remembered name-face pairs used in the name recall task

## Techniques or Strategies for Recalling Names for Faces

Some people are able to use special techniques to help them to remember. Here is a list of memory techniques. We would like to find out the methods that you used. You may have concentrated on the faces and did not do anything else. Or you may have tried many different strategies to try to learn the names. Either way is fine. We are interested only in finding out exactly what you did while you were studying.

**Please place a checkmark by all of the methods that you used while you were studying the names and faces.**

- ☐ **1.** I concentrated and paid attention to the face.
- ☐ **2.** I concentrated and paid attention to the name.
- ☐ **3.** I tried to pick out prominent features.
- ☐ **4.** I tried to think of a meaningful association for the name.
- ☐ **5.** I repeated names over and over to myself.
- ☐ **6.** I tried to think of a concrete object to go with the name, e.g., "Gordon is a garden, Robin is a bird, George is a deep gorge."
- ☐ **7.** I associated the face with the face of someone else I know.
- ☐ **8.** I associated the name with the name of someone else I know.
- ☐ **9.** I made up a sentence that described the person, e.g., "Frank looks like he would be frank and honest – he looks serious."
- ☐ **10.** I made up a sentence that connected the person's name and a prominent facial feature I noticed, e.g., "The red-red-Robin goes bob-bob-bobbin along" (very red cheeks).
- ☐ **11.** I created an image in my mind of the face.
- ☐ **12.** I created an image in my mind of the face with a prominent facial feature exaggerated.
- ☐ **13.** I created an image in my mind of the person with the name written across his or her face.
- ☐ **14.** I created an image in my mind of the person next to an object that fits the name, e.g., "Mr. Kohn with an ice cream cone," "Mr. North with an arrow pointing north."
- ☐ **15.** I used the image-name match method. I created an image in my mind of the person, identified a concrete object related to the name, and I imagined that object next to the person's prominent facial feature.
- ☐ **16.** I covered the faces, looked away and tested myself on the names.
- ☐ **17.** Other method(s): Please describe. ___________________________________

**Now go back through and review the list of things you checked.**
**Circle the 1 or 2 methods that you used the <u>most often</u> for remembering.**

## Techniques or Strategies for Recalling Locations of Objects

Some people are able to use special techniques to help them to remember. Here is a list of memory techniques. We would like to find out the methods that you used. You may have put the objects where you would in your own house and did not do anything else. Or you may have tried many different strategies to try to remember the object locations. Either way is fine. We are interested only in finding out exactly what you did while you were studying.

**Please place a checkmark by all of the methods that you used while you were studying the object locations.**

- ☐ **1.** I concentrated and paid attention to the rooms.
- ☐ **2.** I concentrated and paid attention to the objects.
- ☐ **3.** I concentrated on how the objects looked in the rooms.
- ☐ **4.** I repeated the locations of objects over and over.
- ☐ **5.** I paired the first letter of the object with the first letter of the room.
- ☐ **6.** I put the objects in logical rooms, where most people would expect to find those objects.
- ☐ **7.** I put <u>two</u> objects together in a room because the two objects logically go together (e.g., a lamp and table).
- ☐ **8.** I put <u>individual</u> objects where I put those same objects in my own house.
- ☐ **9.** I put <u>two</u> objects together because I have those objects placed together in the same room in my own house.
- ☐ **10.** I made up a sentence about the objects, one at a time, which matched each object to its room.
- ☐ **11.** I made up a story using many of the objects in their rooms.
- ☐ **12.** I created simple mental images of the objects in the rooms.
- ☐ **13.** I imagined a walk through the "house" seeing each object in each room.
- ☐ **14.** I made active mental pictures, like a movie, that showed the objects in the rooms.
- ☐ **15.** I closed my eyes, thought of the different rooms shown and checked whether I could list the object(s) I placed in each room.
- ☐ **16.** Other method(s): Please describe. ____________________________________

  ______________________________________________________________

  ______________________________________________________________

**Now go back through and review the list of things you checked.
Circle the 1 or 2 methods that you used the <u>most often</u> for remembering.**

## Techniques or Strategies for Recalling Names for Occupations

people are able to use special techniques to help them to remember. Here is a list of memory techniques. We would like to find out the methods that you used. You may have concentrated on the occupations and did not do anything else. Or you may have tried many different strategies to try to learn the names. Either way is fine. We are interested only in finding out exactly what you did while you were studying.

**Please place a checkmark by all of the methods that you used while you were studying the occupations and names.**

☐ **1.** I concentrated and paid attention to the occupation.

☐ **2.** I concentrated and paid attention to the name.

☐ **3.** I repeated names over and over to myself.

☐ **4.** I repeated occupations over and over to myself.

☐ **5.** I repeated names and occupations together.

☐ **6.** I tried to think of a meaningful association for the name.

☐ **7.** I tried to think of a concrete object to go with the name, e.g., “Gordon is a garden. North is a direction.”

☐ **8.** I tried to think about the nature of the occupation, e.g., barbers cut hair with scissors; judges are wise and wear robes.

☐ **9.** I found the same letters in the name and occupation, e.g., “TATE is a TEACHER. WALKER is a BANKER.”

☐ **10.** I associated the occupation with someone I know who has that same occupation.

☐ **11.** I associated the name with the name of someone else I know.

☐ **12.** I made up a sentence that connected the name and occupation, e.g., “Franklin is a professor so he would be frank in his writings.”“Ms. Gor-don (the lawyer) wears a gor-geous suit in court.”

☐ **13.** I created an image in my mind related to the nature of the occupation.

☐ **14.** I created an image in my mind of a person with the occupation, and wrote the paired name across the person’s face.

☐ **15.** I created an image in my mind of a person with the occupation next to a concrete object that fits the name, e.g., “The astronaut Kohn with an ice cream cone.” “The painter North painting an arrow pointing north.”

☐ **16.** I covered the occupations, looked away and tested myself on the names.

☐ **17.** Other method(s). Please describe: ____________________________________

______________________________________________________________________

______________________________________________________________________

**Now go back through and review the list of things you checked.**
**Circle the 1 or 2 methods that you used the <u>most often</u> for remembering.**

**Supplementary Materials D. Transfer Outcome Measures: Object-Location Association Materials**

**Table S1.** List of objects used in the object-location visual association task

| Set A Objects | | Set B Objects | |
|---|---|---|---|
| bottle | legal pad | ashtray | knife |
| box | light bulb | banner | magnifying glass |
| cigars | luggage | baseball | paper bag |
| clock | milk | blender | pen |
| comb | paint bucket | calculator | phone |
| dress | passport | calendar | pillow |
| dumbbell | perfume | camera | purse |
| envelope | radio | cassette tape | screwdriver |
| fire extinguisher | ring | crutches | shoes |
| flag | stapler | football | teapot |
| hole puncher | tennis racket | hand axe | trashcan |
| ladder | thumb tack | horn | wrench |

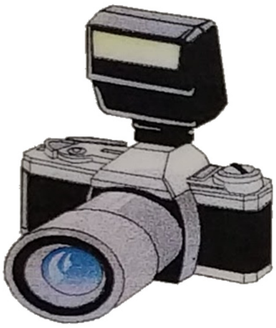

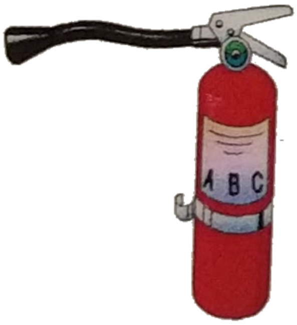


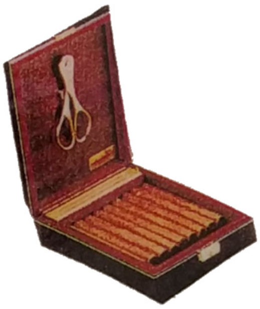

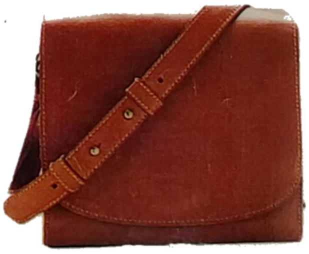

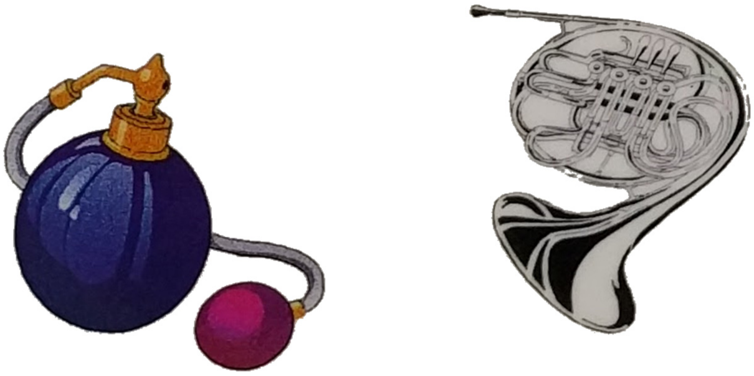

**Figure S2.** Example objects used in the object-location visual association task

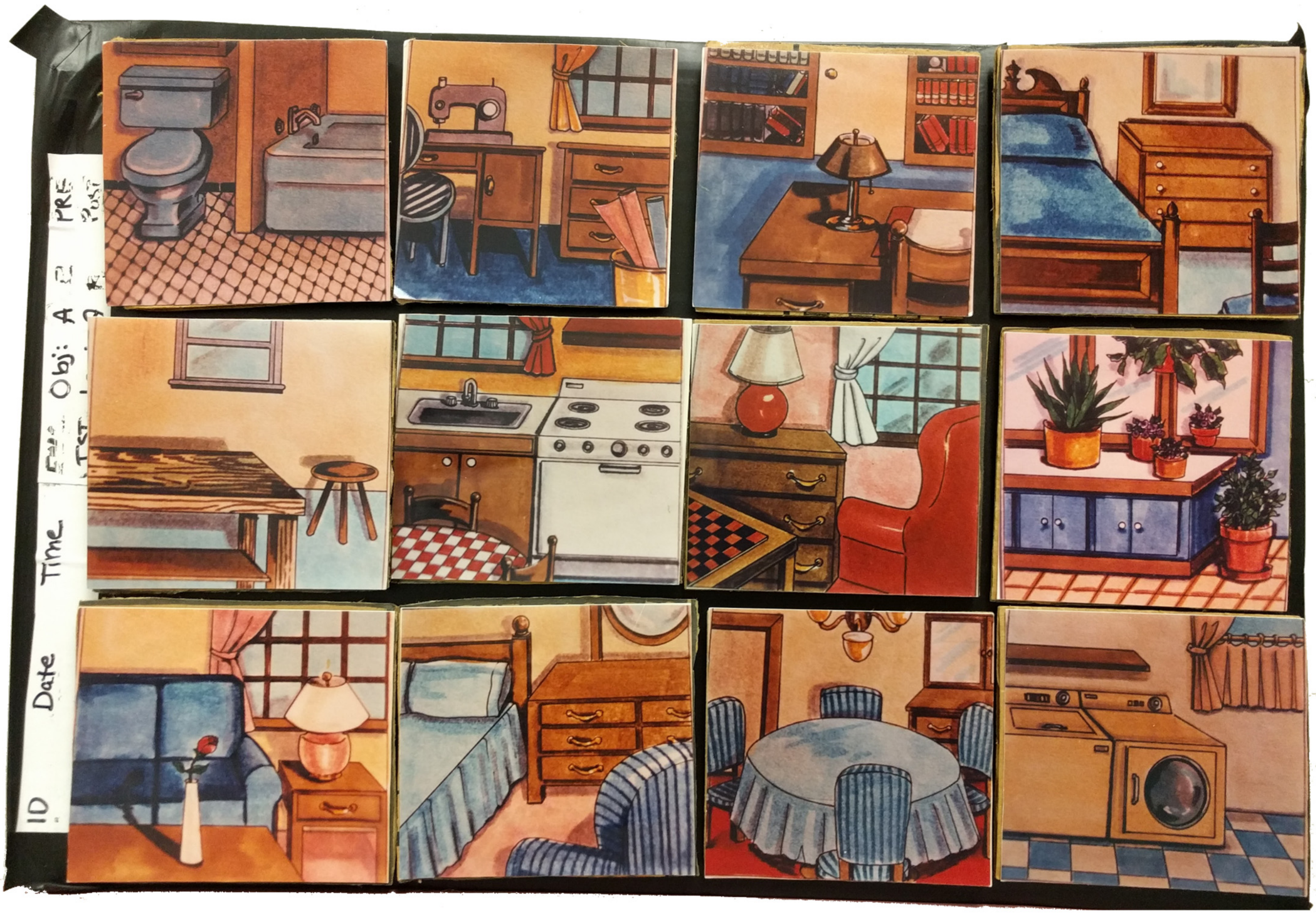


**Figure S3.** Example matrix array used in object-location visual association task

**Supplementary Materials E. Transfer Outcome Measures: Name-Occupation Association Materials**

**Table S2.** List of occupation-name pairs by set

| Set A Pairs | | Set B Pairs | |
|---|---|---|---|
| Occupation | Name | Occupation | Name |
| ARCHITECT | ALLEN | ACCOUNTANT | POTTER |
| CHEMIST | BENNETT | ACTOR | JONES |
| COMIC | OLIVER | ATHLETE | STEVENS |
| CONDUCTOR | WEAVER | BANKER | CARPENTER |
| DENTIST | WAGNER | BUILDER | BANKS |
| DESIGNER | JEFFERSON | BUTCHER | HARRISON |
| DETECTIVE | ROGERS | CLERK | FOX |
| DIPLOMAT | SCOTT | COACH | WATERS |
| DOCTOR | HUNTER | GARDENER | MITCHELL |
| DRIVER | HOWE | GROCER | WILSON |
| ENGINEER | GRAHAM | INVENTOR | MORTON |
| FIREMAN | BUTLER | LOGGER | MONROE |
| GUARD | BISHOP | MANAGER | KING |
| LAWYER | GRAVES | MERCHANT | DIXON |
| LIBRARIAN | SHEPHERD | PILOT | CHURCH |
| MECHANIC | GILMORE | PLUMBER | WELLS |
| MINER | SHOEMAKER | POLICEMAN | DAVIS |
| NURSE | COOK | POLITICIAN | BREWER |
| PAINTER | WILLIAMS | POPE | FLOWERS |
| PASTOR | STANLEY | POSTMAN | GILBERT |
| PRINCE | MORGAN | PRIEST | BARBER |
| PRINTER | WALKER | SALESMAN | SPENCER |
| RABBI | BERRY | SCIENTIST | HARDING |
| REPORTER | BAKER | SERGEANT | HAMILTON |
| RUNNER | STONE | SINGER | FISHER |
| SOLDIER | FLYNN | SURGEON | PETERS |
| TAILOR | DOUGLAS | TUTOR | BROOKS |
| TEACHER | BEARD | WAITER | HOBBS |
| USHER | MEADOWS | WELDER | FARMER |
| VET | CURRY | WRITER | KNIGHT |

**Supplementary Materials F. Training Procedures: Homework Instructions**

**Homework Instructions for Strategy-Only (SO) Training Condition**

# Homework

Please complete all homework before your next interview session or within one week, whichever comes first. Record the date and time spent on each activity on your **Activity Log**.

The purpose of the homework:

1) Show you a wide variety of memory techniques that you could use
2) Help you to raise your scores by using the memory strategies from class

**<u>Assignments</u>**

- ☐ Read ***How Memory Works***
- ☐ Read ***How to Remember Names.***
- ☐ Complete a minimum of 40 minutes of practice with the exercises in the workbook.
- ☐ Find a picture in your home and use Active Observation to remember the details in the picture.
- ☐ Answer the homework questions (below). Please write your answers on the back of this page (use back of Activity Log if you need more space).

1. How are working memory and long-term memory different?

2. How do memory cues work?

3. Use the sentence strategy from the reading on names to remember at least one name this week. Report on how you used the strategy.

4. What kinds of activities do you do at home that are mentally challenging, that require you to think? Write at least two examples.

Do you have any questions about the homework assignments? If so, please contact us by emailing XXX or calling XXX.

**Homework Instructions for Strategy-Plus-Enhanced Beliefs Training Condition**

# Homework

Please complete all homework before your next interview session or within one week, whichever comes first. Record the date and time spent on each activity on your **Activity Log**.

The purpose of the homework:

3) Show you a wide variety of memory techniques that you could use
4) Help you to learn the recommended memory strategies presented in class

**Assignments**

☐ Read ***Age and Memory.***

☐ Read ***How to Remember Names.***

☐ Complete a minimum of 40 minutes of practice with the exercises in the workbook.

☐ Find a picture in your home and use Active Observation to remember the details in the picture.

☐ Answer the homework questions below. Please write your answers on the back of this page (use back of Activity Log if you need more space).

1. Describe at least two age-related memory changes.
2. What should you do if you need more cues to remember?
3. Identify the name learning technique that seems like the best strategy for you. Use this method to remember at least one name this week. Report on how you used the strategy.
4. What kinds of activities do you do at home that are mentally challenging, that require you to think? Write at least two examples.

Do you have any questions about the homework assignments? If so, please contact us by emailing XXX or calling XXX.
